# Predictable and predictive emotions: explaining cheap signals and trust re-extension

**DOI:** 10.3389/fnbeh.2014.00401

**Published:** 2014-11-20

**Authors:** Eric Schniter, Roman M. Sheremeta

**Affiliations:** ^1^Economic Science Institute, Chapman UniversityOrange, CA, USA; ^2^Department of Economics, Weatherhead School of Management, Case Western Reserve UniversityCleveland, OH, USA

**Keywords:** emotion, cheap signal, promise, apology, trust game, reciprocity, experiment

## Abstract

Despite normative predictions from economics and biology, unrelated strangers will often develop the trust necessary to reap gains from one-shot economic exchange opportunities. This appears to be especially true when declared intentions and emotions can be cheaply communicated. Perhaps even more puzzling to economists and biologists is the observation that anonymous and unrelated individuals, known to have breached trust, often make effective use of cheap signals, such as promises and apologies, to encourage trust re-extension. We used a pair of trust games with one-way communication and an emotion survey to investigate the role of emotions in regulating the propensity to message, apologize, re-extend trust, and demonstrate trustworthiness. This design allowed us to observe the endogenous emergence and natural distribution of trust-relevant behaviors, remedial strategies used by promise-breakers, their effects on behavior, and subsequent outcomes. We found that emotions triggered by interaction outcomes are predictable and also predict subsequent apology and trust re-extension. The role of emotions in behavioral regulation helps explain why messages are produced, when they can be trusted, and when trust will be re-extended.

## Introduction

In this paper, we explore the role of positive emotions (pride, believability, appreciation, contentment, cheerfulness, happiness) and negative emotions (guilt, shame, anger, disgust, aggravation, frustration) in regulating cheap signaling, trust re-extension, and trustworthy behavior in the wake of a *veiled* trust-based interaction between strangers with no explicit indication of certain expectation for repeated interaction^1^. Interactions with strangers have presented recurrent adaptive problems over the course of human evolutionary history (Fehr and Henrich, 2003) and are common in modern society, especially in global markets (Nowak and Sigmund, 2005; Seabright, 2010). Sometimes unexpected opportunities for repeated exchange with previously cooperative or uncooperative partners arise^2^. Once exchange histories establish, partners with mutually beneficial non-binding agreements often reap gains from iterated trust-based trade with one another (Cochard et al., 2004; Boero et al., 2009; Kaplan et al., 2012). However, investors ceding resources (in anticipation of desired returns) remain subject to various kinds of exploitation by previously trusted partners. Partners who demonstrated trustworthiness in the past might subsequently engage in Machiavellian manipulations (Humphrey, 1976; Byrne and Whiten, 1988) by sending false signals about intentions to engage in future trustworthy behavior. Upon being re-extended trust, these previously trusted partners can opportunistically exploit their positions^3^. Alternatively, if a partner was previously untrustworthy (e.g., breaking a promise and not returning profits on investment) but claims to have intentions and propensity for future trustworthiness, an investor must decide whether to forgo potential gains from future trust-based exchange (by not re-extending trust) or else pursue the available opportunity with that previously untrustworthy partner, at the risk of being repeatedly deceived or exploited^4^. While much attention has been given to the production and evaluation of cues and signals affecting novel trust extension, less attention has been given to the dilemma of trust re-extension and the role of emotions in regulating relevant behaviors.

The ability to integrate evaluations of reputation from cues and signal quality, infer a partner's propensity toward future trustworthiness, and accordingly regulate trust re-extension would have been a highly advantageous trait over the course of human evolutionary history and should continue to be in modern economies. Evolutionary theories of emotions (Nesse, 1990; Tooby and Cosmides, 1990; Haselton and Ketelaar, 2006; Tooby et al., 2008; Schniter and Shields, 2013) have proposed that key emotions have been selected to assist us in accomplishing these tasks. We test the propositions that new information about trust-based interaction outcomes triggers emotions, and that, when experienced, these emotions regulate re-affirmative and remedial behaviors, and the propensity to re-extend trust.

To study the predictors of emotions and the effects of emotions on spontaneous messaging and trust re-extension, we conducted a non-deceptive study wherein financially motivated participants used endogenously created and naturally distributed promises and messages. Our study is based on a version of the “investment game” by Berg et al. (1995). In our experiments trustees made non-binding promises of investment-contingent returns, then investors decided whether to invest, and finally trustees decided how much to return. Since investing money is risky, investments are usually interpreted as trust. Likewise, because voluntary returns are costly to trustees, the delivery of promised returns on investment (*ROI*) is interpreted as evidence of trustworthiness. We also administered a 20 item survey in which participants reported their emotional status as a consequence of the decisions and interaction outcomes that they just experienced^5^. After an unexpected second game was announced, but before it commenced, trustees could send a one-way message. This design allowed us to observe the endogenous emergence and natural distribution of trust-relevant behaviors, consequent emotions, and focus on these emotions' effects on trustees' naturally occurring communication strategies, investors' trust re-extension, and trustee's trustworthiness.

While results on the observed frequencies of game behaviors in both trust games of this study are reported in the *Journal of Economic Behavior and Organization* (Schniter et al., 2013), we did not previously examine the role of emotions. In this paper we evaluate reports of emotions and their role in regulating behaviors relevant to the dilemma of trust re-extension.

## Background

Despite normative predictions, trust is often developed in experimental one-shot environments with unrelated strangers (Dawes and Thaler, 1988; Kiyonari et al., 2000; McCabe et al., 2003; Krasnow et al., 2013), especially when facilitated by cheap talk and emotions (Frank, 1988, 2004; McElreath et al., 2003; Schweitzer et al., 2006; Ben-Ner and Putterman, 2009; Ben-Ner et al., 2011; Gambetta and Székely, 2014; Sheremeta and Zhang, 2014). In this section we provide some background on three important literatures to which our research links: the first concerns trust-based exchange dilemmas and the evolution of cooperation, the second concerns cheap signaling and trust re-extension, and the third concerns the role of recalibrational emotions in cheap signaling and trust re-extension.

When taking into account the observation that people exist and have long existed under the uncertain but ever present shadow of possible future interactions with others, the propensity to trust (despite hazards for opportunism) may bring net exchange benefits^6^. Under such conditions natural selection may have favored those with the propensity to cooperate even when exposed to indicators that interactions were one-shot and interaction partners were unknown (Delton et al., 2011; Krasnow et al., 2013). Sayings like “you always meet twice in a lifetime,” “you haven't seen the last of me,” and “dangerous enemies will meet again in narrow streets” seem to provide justification for the human tendency to treat others as if they will be beneficial exchange partners or threats in the future, despite the absence of cues assuring there will be repeated interaction. The one-shot investment game (Berg et al., 1995) that models the opportunity to develop and allocate gains through exchange, has shown time and again that people exposed to one-shot sequential exchange opportunities with anonymous others tend to behave in a mutually beneficial way (for reviews see Ostrom and Walker, 2005; Balliet and Van Lange, 2012) despite the normative proscription from game theory: do not cooperate because your partner will not cooperate.

In the absence of information about past behavior, “cheap” messages (bearing little in the way of up-front costs for production) are often sent to receivers with the intention of communicating information about the sender (e.g., see Farrell and Rabin, 1996). For example, non-binding promises (of intended trustworthiness) have been shown to increase cooperation (Rubin and Brown, 1975; Kerr and Kaufman-Gilliland, 1994; Ellingsen and Johannesson, 2004; Charness and Dufwenberg, 2006; Sutter, 2009). Where demonstrated behavior has informed investors of a trustee's untrustworthiness, messages may be sent with the intention of persuading investors that the trustee is more trustworthy than inferred from cues alone. Many find it quite puzzling that so-called “cheap signals” can effectively be used to negotiate trust between individuals with conflicting interests (Lachmann et al., 2001) and that it is even possible for promise-breakers to rebuild damaged trust by issuing apologies (Schniter et al., 2013). Below we review why trust can be built with the help of cheap-to-produce messages and why those who re-extend trust to previously untrustworthy individuals (e.g., to promise breakers) may take their messages into consideration.

Cheap-to-produce messages can maintain their reliability because they often end up being “costly” after being used to deceive (Schniter et al., 2013). Through reputational sanctions or exclusion from future interactions, receivers of deceptively used cheap signals can impose ex post costs greater than the benefits initially derived from deceptively using those signals (Rohwer, 1977; Masclet et al., 2003; Schweitzer et al., 2006)—thereby maintaining signal reliability in the society in which it was used.

Though it has been studied little, it is reasonable to expect that the psychological machinery designed to produce and evaluate cheap signals is attuned to the experience and perception of emotions. Emotions are important components of message composition, speech production and perception, and face-to-face communication. Personal communication in various forms is known to improve cooperation (Orbell et al., 1988; Bohnet and Frey, 1999; Ridings et al., 2002; Zheng et al., 2002; Buchan et al., 2006; Cason et al., 2012) by facilitating coordination, decreasing social distance, raising solidarity, and providing the cues of familiarity that are normally associated with trustworthy relationships. Smith (1759) wrote of the “fellow feeling” that can be generated, for example as a consequence of sharing in another's emotional state, and being part of the process of improving it. According to Smith, the capacity to experience the pleasurable “fellow feeling” is based on our ability to model another's circumstances and emotional reaction to them, and to internally simulate (sympathize with) the emotional feelings that they might derive. When messages are produced^7^, they are often assembled with verbiage meant to demonstrate regard for the recipient, persuade a change in the recipient's perspectives, and provide information of the signaler's intentions and emotional experiences: things which may not be otherwise known (Pennebaker and Graybeal, 2001). Though the messages we consider guarantee no honest information, their length is a potential indication of the effort invested into an attempt to communicate these potentially unknown things. On the other hand, where messages are intended as re-affirmations of known things (e.g., recent cooperation), their length is not as necessary. Thus, to understand the human ecology of cheap signal production and evaluation, one should also understand the dynamic triggering of emotions and their targeted effects on the propensity to engage in communication.

According to the recalibrational theory of emotions, when evolved psychological machinery has computationally identified adaptive problems (such as resulting from social dilemma outcomes), emotional responses are triggered that encourage recalibration of behavior regulation programs (Tooby et al., 2008; Schniter and Shields, 2013; Schniter et al., 2014). As such, emotions are hedonic components of a learning system that integrates relevant experiences to inform individual decisions and interaction behaviors. Emotions are often relatively “automatic,” difficult to control, and distinct from cogitative non-emotional learning^8^. When triggered, emotional responses typically produce sudden physiological changes (e.g., arousal) and affect facial expression, posture, subjective experience and perception, and action tendencies (Frijda, 1986; Tooby and Cosmides, 1990).

Consistent with recalibrational theories of emotions, we hypothesize that emotions integrate new information about trust-based interaction outcomes, providing hedonic feedback that people experience as either *positively* valenced (pleasantly motivating continuation of prior behaviors associated with its occurrence) or *negatively* valenced (unpleasantly motivating disengagement and pursuit of alternative strategies). It is hypothesized that this positive or negative emotional feedback is designed to motivate changes to behavioral propensities so as to enhance success in future relationships characterized by similar cooperation dilemmas. For example, when a trust-based relationship has been developed and assured, good feelings such as *pride* and *appreciation* are experienced (Schniter and Shields, 2013; Schniter et al., 2014). As a result, proud trustees may be more inclined to re-affirm the good relationship and demonstrate more trustworthiness (Nesse, 1990; Fessler, 1999, 2001), and appreciative or grateful investors may be more likely to trust the trustee's cheap signals and re-extend trust (Hirshleifer, 1987; McCullough et al., 2001; Dunn and Schweitzer, 2005; Algoe et al., 2008; Tooby and Cosmides, 2008). Alternatively, when a trustee has demonstrated untrustworthy behavior (breaking a promise or exploiting an investor) the investor may experience anger and frustration (e.g., see Ortony et al., 1988; Dunn and Schweitzer, 2005) while the trustee experiences guilt, and shame (e.g., see Baumeister et al., 1994; Smith et al., 2002; Ketelaar and Au, 2003; Sznycer, 2010; Sznycer et al., 2012). Angry and frustrated investor's may be more likely to distrust subsequent promises or offers from the untrustworthy trustee (e.g., see Pillutla and Murnighan, 1996; Dunn and Schweitzer, 2005), and more likely to impose costs or restrict benefits (e.g., see Sell et al., 2009). A guilty or shameful trustee may be motivated to make remedial efforts (e.g., issuing a persuasive message or apology) targeting the affected investor—especially when there is possibility of future trust-based exchange opportunity with this potentially angry investor (Fessler, 2001; Sznycer, 2010; de Hooge et al., 2011; Sznycer et al., 2012). Apology has been demonstrated to be a particularly effective remedial signal that encourages a victim to trust again by expressing responsibility for an offense and possibly the promise of forbearance, an offer of condolence, or repair (Scher and Darley, 1997; Ho, 2012; Fischbacher and Utikal, 2013; Schniter et al., 2013).

Emotionally affected speech and facial expression have long been believed to have some “honest signal” features revealing underlying emotional states (Darwin, 1872/1965). A century after Darwin, researchers began making a strong case that basic human emotions are not only universal in their distinct facial and vocal expressions, but also that the identification of these emotions exists and is consistent across humans. Evidently, humans can accurately recognize many basic emotions (e.g., happiness, surprise, sadness, fear, disgust, and anger) across quite different cultures in faces (Ekman et al., 1969), voices (Bryant and Barrett, 2008; Sauter et al., 2010), and written messages (Xiao and Houser, 2005) supporting the notion that these communicative forms provide fairly reliable indicators of the positive and negative affect, if not actual emotion, that the sender experiences (Keltner and Kring, 1998) and that a reduction in ambiguity has been selected for in human emotional signaling (Searcy and Nowicki, 2005). Hirshleifer (1987) and Frank (1988, 2004) proposed that emotions provide information about people's behavior propensities (e.g., as guarantors of promises) because they work as commitment devices. As veridical signals, emotions appear to be sufficiently reliable in this capacity; emotional displays are more often than not involuntary and high levels of emotional expressivity are difficult to imitate (Boone and Buck, 2003; Schug et al., 2010). Thus, the standard human ecology in which “cheap” signal production and evaluation was designed to operate is not entirely costless. Rather, because the signaling of emotional states is often veridical, language should be evaluated and trusted according to its correspondence with emotional propensities. Under these conditions, detection of deceptive language production would lead to the imposition of more costs than benefits on the signaler—effectively making cheaply produced language in the context of displayed emotions a “costly signal.”

We anticipated that, despite the anonymity we guaranteed participants in our laboratory implementation of the trust game (i.e., ensuring that their partners would not personally identify or watch them), experiences of emotions would be reliably produced and recognized by those experiencing them^9^. We evaluate the reported experience of several emotions (appreciation, contentment, cheerfulness, happiness, pride, believability, anger, disgust, aggravation, frustration, guilt, and shame) and whether these emotions predict the use of cheaply produced messages, trust re-extension, and trustworthy or opportunistic behavior. We hypothesize that these emotions serve the recalibrational functions outlined in Schniter and Shields (2013), and below detail specific predictions about the triggering of these emotions and how experienced emotions will correlate with subsequent behaviors. Previous studies have shown that the experience of emotions affects subsequent game behavior (Pillutla and Murnighan, 1996; Fehr and Gächter, 2002; Ketelaar and Au, 2003; Capra, 2004; Dunn and Schweitzer, 2005; Hopfensitz and Reuben, 2009; Kausel and Connolly, 2014). The current study contributes to this literature by examining how trust-relevant integral emotions are naturally triggered by interaction outcomes and how they inform trust repair and re-extension in subsequent interactions after trust is damaged^10^.

## Experiment details and hypotheses

### Experiment details

Our research was approved by Chapman University's internal review board for research with human subjects and informed consent was obtained from all participants. The research was conducted at Chapman University's ESI laboratory. 458 participants (229 pairs) were recruited from a standard campus-wide subject pool for participation in a session that could last up to 45 min. There were 25 sessions. Each session had between 10 and 24 participants. The average earnings from experiments were $18, ranging from a $0 to $40, plus $7 for arriving to the session on time and participating. No participant participated more than once, and no participant had prior experience with a similar game environment. During a session, participants seated at visually isolated cubicles interacted with each other anonymously over a local computer network. Our procedure consisting of three parts, lasted an average of 35 min total, did not involve deception^11^, and proceeded as follows. Upon arrival, participants were told that they would receive $7 for participation, to be paid at the end of the session along with any additional money made during the session.

In the first part of the session, participants received instructions (see Supplementary Material) for and interacted in an “experiment”: a *veiled* trust game with (i) no indication of a subsequent game to follow and (ii) no statements that the session would end at conclusion of that game. The first trust game is denoted as “veiled” because participants are intentionally not informed of a subsequent “repetition of the experiment”: a trust game (repeated with same roles and partners as before) that would follow in part three^12^. Participants were assigned to one of two roles: “Participant A” (investor), or “Participant B” (trustee). First, the trustee completed the following standardized statement (which we will refer to below as a promise) by selecting an integer from 0 to 20: “I (Participant B) promise to transfer back $___of my income to you (Participant A) if you choose IN.” This statement was not binding, however. That is, the trustee was not obligated to transfer back the amount promised to the investor, and both trustee and investor knew this. The computer conveyed the trustee's statement to the investor and then the investor chose either OUT or IN. If the investor chose OUT, she received $5 and the trustee $0. If the investor chose IN (invest), then the trustee received $20 (the “income”), after which he selected a whole dollar amount from $0 to $20 to send back to the investor.

In the second part of the session, after the veiled trust game (game 1) finished, participants were given an emotional status survey (see Supplementary Material) that asked them to report how much they felt each of 20 emotional states (on a five point scale labeled (1) very slightly or not at all, (2) a little, (3) moderately, (4) quite a bit, (5) extremely) as a consequence of their recent game interactions and outcomes^13^. The computer software presented all emotional states on one screen and in random order.

In the third part of the session, after completing the survey, we gave our participants additional instructions (see Supplementary Material) indicating that they had opportunity to participate in a “repetition of the experiment.” These instructions unveiled that in game 2, participants would remain in the same roles and interact with the same partner as in game 1. The instructions also indicated that prior to game 2, the trustee would have an opportunity to use a “message” box to send a one-way message to the investor. Trustees were told that “in these messages, no one is allowed to identify him or herself by name, number, gender, or appearance,” but that other than these restrictions, trustees could “say anything in the message.” If trustees wished not to send a message they were instructed to “simply click on the send button without having typed anything in the message box.” The computer conveyed the trustee's message and subsequently the standardized promise to the investor, and then game 2 proceeded. We specified that game 2, which had the same rules as game 1, was the last experimental game in the session.

We classified whether messages from our study were *apology* (or not) using an incentivized laboratory coordination game (Houser and Xiao, 2011). Three coders recruited from the subject pool and blind to the hypotheses^14^ were asked to code each message based on whether or not it conformed to a broad definition of apology (“an explicit or implicit acknowledgment of offense”). All messages without content were coded by all coders as not conforming to the definition of apology and 93% of messages with content were coded by the majority of coders as conforming to the definition, a “substantial” inter-coder agreement (Kappa of 0.70)^15^.

### Hypotheses

An earlier publication in the *Journal of Economic Behavior and Organization* (Schniter et al., 2013) detailed behavioral results from games 1 and 2 of this study, with particular attention to the effects of promises, transfers, messages, and apologies. This earlier publication did not examine the role of emotion experiences as a result of these games and gave less attention to the unanticipated dilemma of trust re-extension that we focus on here.

When deciding whether to re-extend trust, it is important for an investor to obtain accurate information about the propensity of trustees to behave in a trustworthy manner. The integrity of a trustee's previous promise (i.e., its *signal value*) and the actual returns made on investment (*ROI*) are indicators of trustworthy character demonstrated in past word and action or action alone, respectively. Previous studies suggest that these indicators of trustworthiness, with and without verbal components, may have separate effects on subsequent trust extension (Schweitzer et al., 2006; Schniter et al., 2013), so we examine their effects separately ^16^. We hypothesize that these demonstrations of trustworthy character trigger emotional reactions in investors and trustees facing particular problems and that the emotions better calibrate them for repeated interaction with one another.

Specifically, we predict that when the *signal value* (= return − promise) of trustworthiness is negative (≥0), the trustee's psychology implicitly recognizes potential for a subsequent *promise breaker*'s (*promise keeper*'s) cooperation problem and triggers an emotional reaction: lower (higher) levels of pride and believability, higher (lower) levels of guilt and shame. When the non-verbal indicator of trustworthiness, *ROI* (= return/investment), is greater (not greater) than one, the trustee implicitly recognizes potential for a subsequent *beneficent* (*opportunist*) cooperation problem and an emotional reaction is triggered: feeling higher (lower) levels of pride and believability, lower (higher) levels of guilt and shame. We expect that together the trustee's *beneficent* (*opportunist*) and *promise breaker* (*promise keeper*) emotional reactions inform the trustee's propensity to produce spontaneous re-affirmative or remedial behaviors (constructing *messages* with content, constructing wordier messages with higher *word count*, and issuing spontaneous *apologies*) in preparation for a subsequent interaction problems.

We predict that when the *signal value* (= return − promise) is negative (positive), the investor implicitly recognizes potential for a subsequent *damaged trust* (*assured trust*) cooperation problem and an emotional reaction is triggered, characterized by higher (lower) levels of anger, disgust, aggravation, frustration, and lower (higher) levels of appreciation, contentment, cheerfulness, and happiness. When *ROI* is greater than one (one or less), the investor experiences a *benefited* (*exploited*) emotional reaction: feeling lower (higher) levels of anger, disgust, aggravation, frustration, and higher (lower) levels of appreciation, contentment, cheerfulness, and happiness.

**Figure 1:**
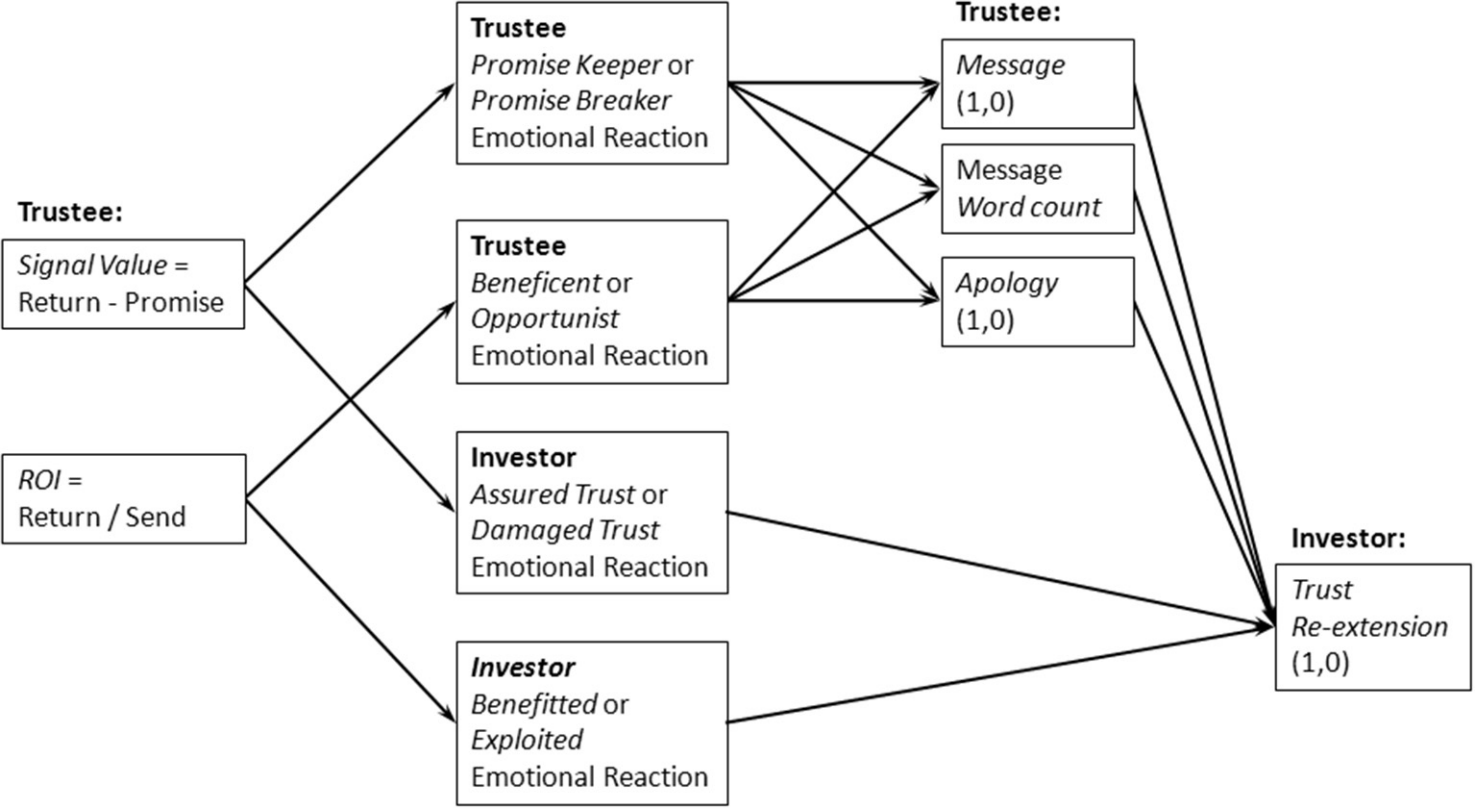
**Path model of predicted relationship between variables**.

We expect that the propensity to re-extend trust in game 2 is informed by the investor's emotional reactions to demonstrated trustworthiness, and some assessment of re-affirmative and remedial messaging (e.g., whether or not there is a *message* with content, what the *word count* of message is, whether or not an *apology* was issued) by the trustee after the investor's initial emotional reactions to game 1 interaction outcomes^17^. In Figure 1 we provide a path model visually representing the above predicted relationships between indicators of trustworthiness, emotional reactions to cooperation problems resulting from game 1 interaction outcomes, messaging behaviors, and the trust of re-extension decision. We suspect that investor emotional reactions to trustee re-affirmative and remedial behaviors would also affect our model, however we did not survey emotional reactions to either received messages or game 2 promises and are therefore unable to account for their partial effects.

In addition to the predicted emotional effects on messaging and trust re-extension summarized above and in Figure 1, we predict emotional effects on trustee behavior in game 2. Specifically, we predict a greater propensity to generate trustworthy indicators (e.g., with higher *signal value* and higher *ROI*) in game 2 among trustees who reported higher levels of emotional reaction to game 1—regardless of reaction valence (i.e., higher levels of feeling proud, believable, guilty, and ashamed). Conversely, we expect those reporting less emotional reaction to demonstrate less trustworthy behaviors.

## Results

Trust and reciprocity decisions from this game are reported in detail in Schniter et al. (2013); however, the role of game outcomes in triggering emotional experiences, and the role of emotional experiences in affecting subsequent behavior propensity was not reported.

### General overview

We found no significant differences between the 25 sessions and report the joint results of all 458 participants where appropriate. As seen with similar games, we observed a high initial rate of promised cooperation: 95.2% (218/229) of trustees promised investors *ROI* > 1, 3.9% (9/229) promised *ROI* = 1, and 0.8% (2/229) promised *ROI* < 1. In game 1, there was also a high rate of trust in response to the promises: investors trusted 86.7% (189/218) of trustees promising *ROI* > 1 and 22.2% (2/9) promising *ROI* = 1, but none of those promising *ROI* < 1. Trusting investors from the first game (83.4% of all investors) faced a new set of challenges when they interacted with the same trustees again in a second unexpected game. Some of these investors decided whether to re-extend trust to trustees who cooperated in the first game by delivering a *ROI* > 1 (which 88.5% did), delivering the returns they promised (which 81.2% did), or both. Other investors decided whether to re-extend trust to trustees that did not deliver the returns they promised (18.8% of all trusted trustees), or did not deliver a profitable *ROI* (11.5% of all trusted trustees), or both. A subset of those who decided whether to re-extend trust to non-cooperators, appear to have also been influenced by their emotional reactions to game 1 and subsequent message and apology that were sometimes received.

We examined emotional state reports from the 382 participants who had game 1 interactions where trust was extended. We found moderately high reliability of internal consistency in their emotion reports: the Cronbach alpha coefficient is 0.889 for the 10 item Positive Affect Scale and 0.888 for the 10 item Negative Affect Scale of our 20 item survey, a version of the PANAS (Watson et al., 1988)^18^. There was substantial variability in individual reports of the 12 emotional states investigated below^19^. The average reported emotional state had a mean of 2.21 (median = 1, *SD* = 1.04), near 2 (“a little”). Ratings on every emotional state ranged from 1 (“very slightly or not at all”) to 5 (“extremely”). While the modal report for most (7/12) emotional states was 1 (“very slightly or not at all”) modes were also seen at 3 for believable and 4 for appreciative, content, cheerful, and happy. Reports of 1 were more frequent for emotional states in the negative set than for the positive set (1968/2292 vs. 400/2292, respectively), contributing to significantly lower intensity of reported negative states (*M* = 1.27, *SD* = 0.75) than positive states (*M* = 3.15, *SD* = 1.34) according to Wilcoxon matched-pairs tests (*Z* = −15.167, *p* < 0.001). This pattern of significantly lower reported negative states was observed in both investors (*Z* = −9.446, *p* < 0.001) and trustees (*Z* = −11.798, *p* < 0.001).

In this paper, we focus on the explanatory power of the emotional reactions we predict based on recalibrational theory. Below we report results indicating the predictable and predictive nature of emotional reactions in this unexpectedly repeated interaction. Emotions help explain whether participants attempted to use cheap signaling reassuringly or remedially, whether investors decided to re-extend trust, and whether individuals were more likely to break promises or benefit their exchange partners.

### Predicted emotions

#### Trustees: emotions predicted by behavior

In this section we evaluate whether the positive and negative emotional reactions to game 1, reported by naturally occurring groups of trustees and investors, were predicted by demonstrations of trustworthiness (*signal value, ROI*) after being trusted (i.e., invested in). As predicted, after game 1, *promise breakers* reported significantly higher levels of guilt and shame than *promise keepers*, and *promise keepers* reported significantly higher levels of pride and feeling believable than *promise breakers*. We provide bar charts of *promise breaker*'s and *promise keeper*'s emotions and report the details of Welch's *t*-test comparisons for equal means in Supplementary Material.

Also, consistent with our prediction of a *beneficent* (*opportunist*) emotional reaction in trustees, *beneficent* trustees delivering *ROI* > 1 in game 1 reported significantly higher levels of pride and feeling believable. *Opportunist* trustees reported significantly higher levels of guilt and shame. We provide bar charts of *beneficent* and *opportunist* trustees' emotions and report the details of Welch's *t*-test comparisons in Supplementary Material.

**Figure 2:**
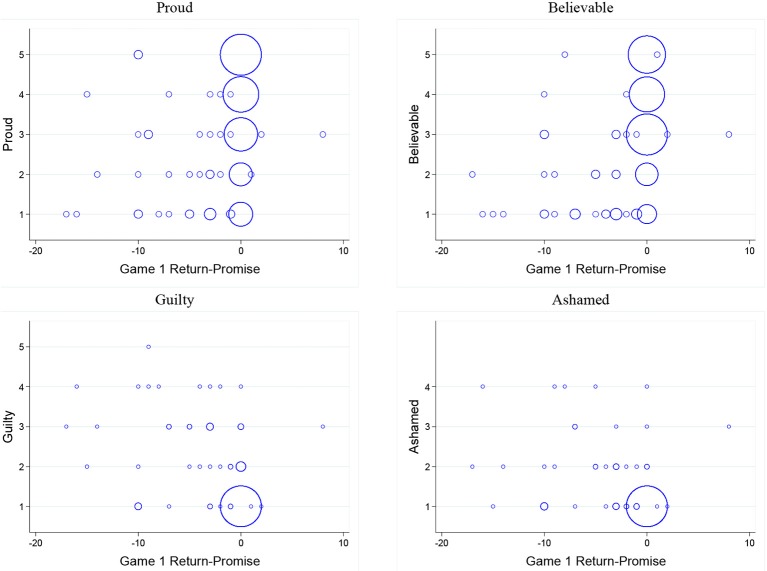
**Bubble plots of trustees' emotions and *Signal value***.

Figure 2 displays bubble plots of trustee emotion intensity (on a five point scale) and *signal value* (the difference between game 1 return and promise). Observations are plotted with bubbles, where the relative size indicates the proportion of observations at a given point. Table 1 reports results of linear regression models where the dependent variable is intensity of a specific emotion (proud, believable, guilty, ashamed) and the independent variable is *signal value*. We find that trustee emotions are predicted by *signal value* of the promise.

**Table 1:** **Regression of trustees' emotions on *signal value***.

**Dependent variable**	**Proud**	**Believable**	**Guilty**	**Ashamed**
**Specification**	**(1)**	**(2)**	**(3)**	**(4)**
Game 1 return-promise	0.116^***^	0.164^***^	−0.138^***^	−0.084^***^
	(0.031)	(0.028)	(0.016)	(0.012)
Constant	3.429^***^	3.394^***^	1.222^***^	1.110^***^
	(0.105)	(0.096)	(0.055)	(0.042)
*R*-squared	0.070	0.151	0.280	0.193
Observations	191	191	191	191

*** *Significant at 1%*.

*Standard errors in parenthesis*.

**Figure 3:**
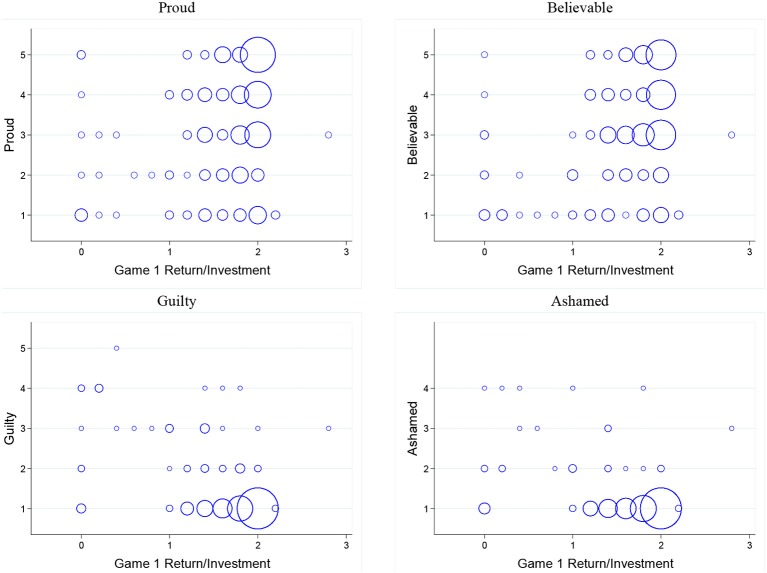
**Bubble plots of trustees' emotions and *ROI***.

Similarly, Figure 3 displays bubble plots of trustee emotion intensity and *ROI*. Table 2 reports results of estimating linear regression models where the dependent variable is intensity of a specific emotion (proud, believable, guilty, ashamed) and the independent variable is *ROI*. We find that trustee emotions are predicted by *ROI*. This leads us to our first result:

**Table 2:** **Regression of trustees' emotions on *ROI***.

**Dependent variable**	**Proud**	**Believable**	**Guilty**	**Ashamed**
**Specification**	**(1)**	**(2)**	**(3)**	**(4)**
Game 1 return/investment	0.709^***^	0.872^***^	−0.821^***^	−0.481^***^
	(0.185)	(0.173)	(0.097)	(0.076)
Constant	2.137^***^	1.781^***^	2.722^***^	1.992^***^
	(0.319)	(0.298)	(0.167)	(0.130)
*R*-squared	0.072	0.119	0.275	0.176
Observations	191	191	191	191

*** *Significant at 1%*.

*Standard errors in parenthesis*.

**Result 1:** Trustees' demonstrations of trustworthiness in game 1, by *signal value* of promise and *ROI*, predict their subsequent emotions.

#### Investors: emotions predicted by behavior

As predicted, investors with *damaged trust* (where game 1 return < promise) reported significantly higher levels of anger, disgust, aggravation, and frustration while investors who had been *assured* with a return ≥ promise reported significantly higher levels of appreciation, contentment, cheerfulness, and happiness. We provide bar charts of emotions reported by investors with *damaged trust* and *assured trust* and report the details of Welch's *t*-test comparisons in Supplementary Material.

Also, consistent with our prediction, significantly lower levels of anger, disgust, aggravation, frustration, and significantly higher levels of appreciation, contentment, cheerfulness, and happiness were reported after game 1 by investors who had *benefited* from a *ROI* > 1, as opposed to those *exploited* by lower *ROI*. We provide bar charts of emotions reported by investors who had *benefited* and been *exploited* and report the details of Welch's *t*-test comparisons in Supplementary Material.

**Figure 4:**
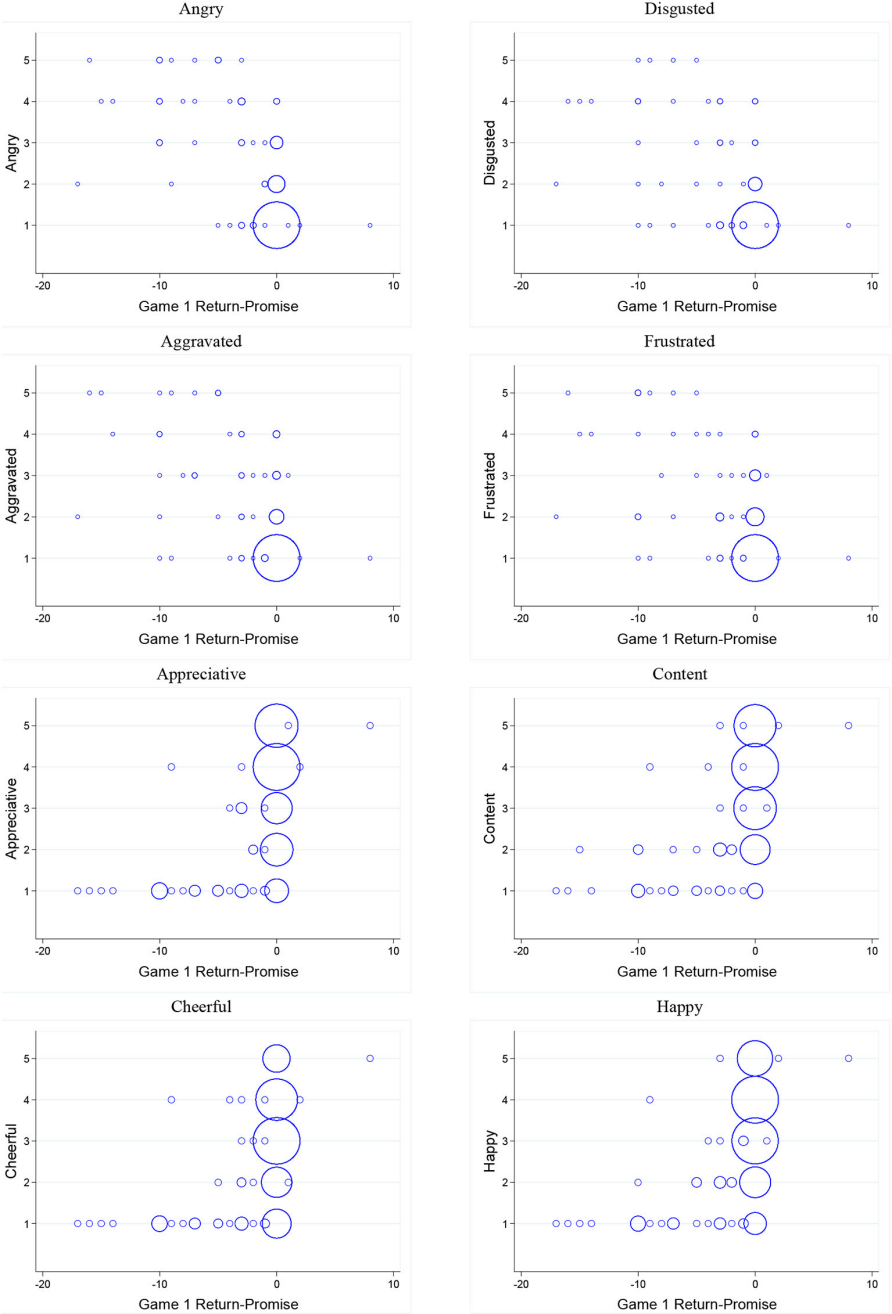
**Bubble plots of investors' emotions and *Signal value***.

Figure 4 display bubble plots of investor emotion intensity and *signal value* of promise. Observations are plotted with bubbles, where the relative size indicates the proportion of observations at a given point. Table 3 reports results of estimating linear regression models where the dependent variable is intensity of a specific emotion (angry, disgusted, aggravated, frustrated, appreciative, content, cheerful, happy) and the independent variable is *signal value*. We find that investor emotions are predicted by *signal value* of promise.

**Table 3:** **Regression of investors' emotions on *signal value***.

**Dependent variable**	**Angry**	**Disgusted**	**Aggravated**	**Frustrated**	**Appreciative**	**Content**	**Cheerful**	**Happy**
**Specification**	**(1)**	**(2)**	**(3)**	**(4)**	**(5)**	**(6)**	**(7)**	**(8)**
Game 1 return-promise	−0.232^***^	−0.188^***^	−0.208^***^	−0.193^***^	0.227^***^	0.209^***^	0.181^***^	0.202^***^
	(0.019)	(0.017)	(0.019)	(0.018)	(0.029)	(0.026)	(0.026)	(0.026)
Constant	1.372^***^	1.212^***^	1.290^***^	1.338^***^	3.455^***^	3.550^***^	3.031^***^	3.296^***^
	(0.066)	(0.057)	(0.063)	(0.063)	(0.098)	(0.087)	(0.090)	(0.088)
*R*-squared	0.432	0.397	0.400	0.365	0.248	0.263	0.200	0.244
Observations	191	191	191	191	191	191	191	191

*** *Significant at 1%*.

*Standard errors in parenthesis*.

**Figure 5:**
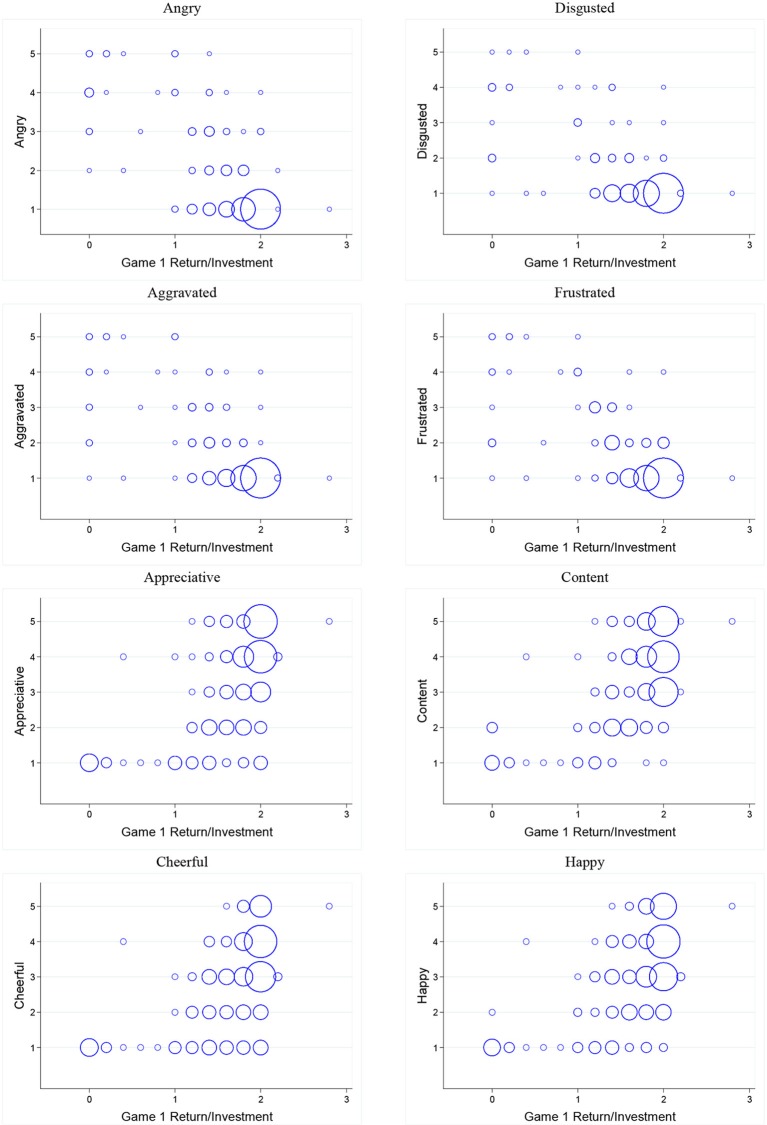
**Bubble plots of investors' emotions and *ROI***.

Similarly, Figure 5 displays bubble plots of investor emotion intensity and *ROI*. Table 4 reports results of estimating linear regression models where the dependent variable is intensity of a specific emotion (angry, disgusted, aggravated, frustrated, appreciative, content, cheerful, happy) and the independent variable is *ROI*. We find that investor emotions are predicted by *ROI*. This leads us to our second result:

**Table 4:** **Regression of investors' emotions on *ROI***.

**Dependent variable**	**Angry**	**Disgusted**	**Aggravated**	**Frustrated**	**Appreciative**	**Content**	**Cheerful**	**Happy**
**Specification**	**(1)**	**(2)**	**(3)**	**(4)**	**(5)**	**(6)**	**(7)**	**(8)**
Game 1 return/investment	−1.508^***^	−1.171^***^	−1.346^***^	−1.324^***^	1.615^***^	1.497^***^	1.366^***^	1.422^***^
	(0.109)	(0.099)	(0.105)	(0.101)	(0.161)	(0.142)	(0.147)	(0.146)
Constant	4.104^***^	3.341^***^	3.728^***^	3.724^***^	0.553^**^	0.863^***^	0.590^**^	0.738^***^
	(0.187)	(0.170)	(0.181)	(0.174)	(0.278)	(0.244)	(0.254)	(0.251)
*R*-squared	0.505	0.425	0.464	0.477	0.346	0.371	0.313	0.334
Observations	191	191	191	191	191	191	191	191

** *Significant at 5%*.

*** *Significant at 1%*.

*Standard errors in parenthesis. Constants are not reported*.

**Result 2:** Trustees' demonstrations of trustworthiness in game 1, by *signal value* of promise and *ROI*, predict investors' subsequent emotions.

### Predictive emotions

#### Trustees: spontaneous messaging behaviors predicted by emotions

Using regression analysis, we evaluated the effects of trustees' emotional reactions to game 1 on measures of their spontaneous re-affirmative and remedial behaviors (sending a message with content, the word count sent, and inclusion of a spontaneous apology). Table 5 reports how the likelihood of *message* (i.e., whether or not they sent a message with content) depends on different emotions. We also controlled for the trustee *signal value*, as a broken promise may have been a prime motivator for sending messages with content. Nevertheless, trustees' feelings of pride showed a significant positive relationship predicting *message* (*p* = 0.027), explaining 2.7% of variance in *message*. Trustees feeling believable, guilt, or ashamed were not predictive of *message*.

**Table 5:** **Regression of *message* on trustees' emotions**.

**Dependent variable**	**Message**	**Message**	**Message**	**Message**	**Message**
**Specification**	**(1)**	**(2)**	**(3)**	**(4)**	**(5)**
Proud	0.300^**^				0.330^**^
	(0.136)				(0.146)
Believable		0.184			0.120
		(0.147)			(0.156)
Guilty			0.344		0.562
			(0.310)		(0.429)
Ashamed				0.291	0.029
				(0.402)	(0.539)
Game 1	−0.052	−0.046	0.028	0.006	0.003
return-promise	(0.064)	(0.066)	(0.070)	(0.066)	(0.076)
Constant	0.429	0.800	1.002^**^	1.090^**^	−0.761
	(0.465)	(0.513)	(0.405)	(0.474)	(0.845)
*R*-squared	0.027	0.009	0.008	0.004	0.047
Observations	191	191	191	191	191

** *Significant at 5%*.

*Standard errors in parenthesis*.

Table 6 reports how the message *word count* depends on different emotions, as well as the trustee *signal value*. We used a hurdle model, described by Cameron and Trivedi (1998), since the process of generating zero values (i.e., no words) is likely to be different from the process of generating positive values. Trustees' pride showed a significant negative relationship (*p* < 0.001), while feeling believable as well as feelings of guilt and shame showed a significant positive relationship (*p* = 0.010, *p* < 0.001 and *p* < 0.001), with *word count*. Next, we estimated a regression to evaluate the combined effects of the above four emotional reactions on message *word count*. We can reject the hypothesis that all four emotions (specification 5 in Table 6) have no effect on *word count* (*X*^2^ = 62.20, *p* < 0.001, *df* = 4). As a set, the four emotions predicted 5.5% of the variance in message *word count*.

**Table 6:** **Regression of *word count* on trustees' emotions**.

**Dependent variable**	**Word count**	**Word count**	**Word count**	**Word count**	**Word count**
**Specification**	**(1)**	**(2)**	**(3)**	**(4)**	**(5)**
Proud	−0.046^***^				−0.040^**^
	(0.015)				(0.017)
Believable		0.042^**^			0.081^***^
		(0.016)			(0.017)
Guilty			0.160^***^ (0.024)		0.206^***^ (0.036)
Ashamed				0.132^***^	−0.064
				(0.031)	(0.045)
Game 1 return-promise	−0.035^***^	−0.047^***^	−0.016^**^	−0.028^***^	−0.023^***^
	(0.005)	(0.006)	(0.006)	(0.006)	(0.007)
Constant	2.881^***^	2.575^***^	2.514^***^	2.569^***^	2.386^***^
	(0.057)	(0.061)	(0.038)	(0.042)	(0.093)
*R*-squared	0.029	0.027	0.044	0.032	0.055
Observations	191	191	191	191	191

** *Significant at 5%*.

*** *Significant at 1%*.

*Standard errors in parenthesis*.

Next, Table 7 reports logistic regression analyses evaluating the effects of trustee emotional reactions to game 1 on spontaneous *apology*. Trustees' feelings of pride did not show a significant relationship predicting *apology*. On the other hand, trustees feeling believable showed a significant negative relationship (*p* = 0.019) while feelings of guilt and shame showed a significant positive relationship (*p* = 0.000 and *p* = 0.005) predicting *apology*. Next, we estimated a logit model to evaluate the combined effects of the above four emotional reactions on likelihood of *apology*. We can reject the hypothesis that all four emotions (specification 5 in Table 7) have no effect on the likelihood of apology (*X*^2^ = 19.56, *p* < 0.001, *df* = 4). The Cox and Snell *R*^2^ indicates that together the four emotions explain 41.8% of the *apology* variance.

**Table 7:** **Regression of *apology* on trustees' emotions**.

**Dependent variable**	**Apology**	**Apology**	**Apology**	**Apology**	**Apology**
**Specification**	**(1)**	**(2)**	**(3)**	**(4)**	**(5)**
Proud	−0.243				0.162
	(0.169)				(0.227)
Believable		−0.437^**^			−0.342
		(0.187)			(0.235)
Guilty			1.283^***^		1.371^***^
			(0.293)		(0.420)
Ashamed				1.081^***^	−0.193
				(0.389)	(0.493)
Game 1	−0.407^***^	−0.366^***^	−0.291^***^	−0.346^***^	−0.262^***^
Return-Promise	(0.083)	(0.083)	(0.081)	(0.080)	(0.085)
Constant	−1.535^***^	−0.973^*^	−4.131^***^	−3.576^***^	−3.486^***^
	(0.576)	(0.589)	(0.544)	(0.551)	(1.119)
*R*-squared	0.292	0.312	0.405	0.328	0.418
Observations	191	191	191	191	191

* *Significant at 10%*.

** *Significant at 5%*.

*** *Significant at 1%*.

*Standard errors in parenthesis*.

**Result 3:** Trustees' emotions' predict their subsequent messaging behavior such as issuing *messages* with content, with longer *word count*, and with *apology*.

#### Investors: trust re-extension predicted by emotions

In this section we evaluate whether investors' trust re-extension in game 2 was predicted by their emotional reactions to game 1 and by measures of spontaneous re-affirmative or remedial messaging behavior demonstrated after game 1.

Table 8 reports logistic regression analyses evaluating the effects of trustee emotional reactions to game 1 on *trust re-extension* in game 2. We also include one of several measures of messaging behaviors (i.e., *message, word count*, or *apology*) that investors were targeted by, as well as the trustee *signal value* from game 1 and new *promises* (specifically, the returned amount promised) in game 2. In estimating these models, we do not consider a model with multiple measures of messaging behaviors because it would introduce multicollinearity^20^.

**Table 8:** **Regression of *trust re-extension* on investors' emotions**.

**Dependent variable**	**Game 2 investment**	**Game 2 investment**	**Game 2 investment**	**Game 2 investment**	**Game 2 investment**
**Specification**	**(1)**	**(2)**	**(3)**	**(4)**	**(5)**
Angry	−0.068	−0.113	0.038	0.004	−0.095
	(0.325)	(0.352)	(0.380)	(0.368)	(0.342)
Disgusted	−0.062	−0.002	−0.308	−0.380	−0.230
	(0.360)	(0.386)	(0.415)	(0.441)	(0.418)
Aggravated	0.467	0.709	0.899	0.578	0.507
	(0.516)	(0.566)	(0.599)	(0.566)	0.507
Frustrated	−0.858^*^	−0.929^*^	−0.934^*^	−0.695	−0.829^*^
	(0.471)	(0.490)	(0.516)	(0.491)	(0.504)
Appreciative	0.041	−0.094	−0.077	−0.015	−0.057
	(0.307)	(0.333)	(0.347)	(0.328)	(0.339)
Content	−0.101	−0.088	−0.034	−0.038	−0.016
	(0.321)	(0.327)	(0.353)	(0.335)	(0.344)
Cheerful	0.570	0.526	0.491	0.480	0.545
	(0.375)	(0.382)	(0.403)	(0.385)	(0.395)
Happy	0.013	0.072	0.095	−0.025	−0.019
	(0.416)	(0.431)	(0.436)	(0.439)	(0.432)
Game 1		0.157^*^	0.202^**^	0.165	0.268^*^
return-promise		(0.094)	(0.100)	(0.103)	(0.138)
Game 2		0.171	0.186	0.155	0.198
promise		(0.118)	(0.122)	(0.121)	(0.126)
Message			1.994^***^		
			(0.589)		
Word count				0.053^**^	
				(0.026)	
Apology					2.635^**^
					(1.182)
Constant	1.795	0.386	−1.319	0.167	0.237
	(1.230)	(1.584)	(1.748)	(1.664)	(1.714)
*R*-squared	0.178	0.203	0.288	0.239	0.255
Observations	191	191	191	191	191

* *Significant at 10%*.

** *Significant at 5%*.

*** *Significant at 1%*.

*Standard errors in parenthesis*.

We can reject the hypothesis that all eight emotions in specification (1) of Table 8 have no effect on the likelihood of trust re-extension (*X*^2^ = 21.54, *p* = 0.005, *df* = 8, Cox Snell *R*^2^ = 0.178). Overall, it appears that the most important emotion predicting trust re-extension is the experience of frustration. Specification (2) of Table 8 considers these emotions, as well as the trustee game 1 *signal value* and new *promises* in game 2 to predict trust re-extension (*X*^2^ = 24.06, *p* = 0.007, *df* = 10, Cox Snell *R*^2^ = 0.203). The change in *R*^2^ between specification (2) and specification (1) indicates that *signal value* and new *promises* explain an additional 2.5% of the variance. Specification (3) of Table 8 additionally considers *message* to predict trust re-extension (*X*^2^ = 27.43, *p* = 0.004, *df* = 11, Cox Snell *R*^2^ = 0.288). The change in *R*^2^ between specification (3) and specification (2) indicates that *message* explains an additional 8.5% of the variance. Similar conclusions can be drawn for specification (4) and specification (5).

**Result 4:** Investors' emotions, trustees' messaging targeting investors, and the *signal value* of game 1 promise predict subsequent game 2 *trust re-extension*.

#### Trustees: signal value and ROI in game 2 predicted by emotions

In this section we evaluate whether trustees' emotional reactions to game 1 are predictive of game 2 demonstrations of trustworthiness (*signal value* and *ROI* in game 2).

For previously trusted trustees who were re-extended trust again in game 2, we estimated linear regression models, reported in Tables 9A,B, where the dependent variable is the *signal value* of game 2 promise (the difference between game 2 return and promise) and the independent variables are the trustee emotional reactions to game 1 (pride, believability, guilt, shame), as well as the *signal value* in game 1 (to control for individual effects). Table 9A uses a sub-sample of game 1 promise keepers and Table 9B uses a sub-sample of game 1 promise breakers. There are two interesting results that emerge from comparing these two tables. First, the *signal value* in game 1 and the *signal value* in game 2 are positively correlated for promise breakers (Table 9A) and negatively correlated for promise keepers (Table 9B). Second, it appears that emotions play a more important role in regulating the subsequent behavior of promise keepers than promise breakers. Table 9B shows that trustees feelings of pride in game 1 is positively correlated with *signal value* in game 2, trustees feelings of shame is negatively correlated with *signal value* in game 2.

**Table 9A:** **Regression of game 2 *Signal Value* on promise breaking trustees' emotions**.

**Dependent variable**	**Game 2 return-promise**	**Game 2 return-promise**	**Game 2 return-promise**	**Game 2 return-promise**
**Specification**	**(1)**	**(2)**	**(3)**	**(4)**
Game 1	0.716^***^	0.727^***^	0.766^***^	0.757^***^
return-promise	(0.210)	(0.207)	(0.211)	(0.216)
Proud	−0.609			
	(0.745)			
Believable		−1.089		
		(0.857)		
Guilty			0.943	
			(0.807)	
Ashamed				0.734
				(0.974)
Constant	−1.19	−0.527	−4.580^*^	−3.647
	(2.301)	(2.242)	(2.371)	(2.192)
*R*-squared	0.274	0.294	0.289	0.272
Observations	36	36	36	36

* *Significant at 10%*.

*** *Significant at 1%*.

*Standard errors in parenthesis*.

**Table 9B:** **Regression of game 2 *Signal Value* on promise keeping trustees' emotions**.

**Dependent variable**	**Game 2 return-promise**	**Game 2 return-promise**	**Game 2 return-promise**	**Game 2 return-promise**
**Specification**	**(1)**	**(2)**	**(3)**	**(4)**
Game 1 return-promise	−0.863^**^	−0.887^**^	−0.774^*^	−0.562
	(0.415)	(0.417)	(0.441)	(0.460)
Proud	0.368^*^			
	(0.208)			
Believable		0.244		
		(0.233)		
Guilty			−0.590	
			(0.657)	
Ashamed				−1.507^*^
				(0.884)
Constant	−2.745^***^	−2.303^***^	−0.786	0.131
	(0.787)	(0.871)	(0.779)	(0.962)
*R*-squared	0.049	0.037	0.035	0.048
Observations	155	155	155	155

* *Significant at 10%*.

** *Significant at 5%*.

*** *Significant at 1%*.

*Standard errors in parenthesis*.

Next, we estimated linear regression models, reported in Tables 10A,B, where the dependent variable is game 2 *ROI* (game 2 return divided by investment) and the independent variables are the trustee emotional reactions to game 1, as well as game 1 *ROI* (to control for individual effects). Table 10A uses a sub-sample of game 1 *promise keepers* and Table 10B uses a sub-sample of game 1 *promise breakers*. The only significant emotion predicting *ROI* is shame experienced by *promise keepers* (Table 10B).

**Table 10A:** **Regression of game 2 *ROI* on promise breaking trustees' emotions**.

**Dependent variable**	**Game 2 return/ investment**	**Game 2 return/ investment**	**Game 2 return/ investment**	**Game 2 return/ investment**
**Specification**	**(1)**	**(2)**	**(3)**	**(4)**
Game 1 return-promise	0.720^***^	0.699^***^	0.760^***^	0.759^***^
	(0.233)	(0.233)	(0.237)	(0.238)
Proud	−0.057			
	(0.126)			
Believable		−0.119		
		(0.148)		
Guilty			0.102	
			(0.138)	
Ashamed				0.107
				(0.165)
Constant	0.519	0.624	0.105	0.162
	(0.387)	(0.387)	(0.461)	(0.433)
*R*-squared	0.230	0.241	0.238	0.235
Observations	36	36	36	36

*** *Significant at 1%*.

*Standard errors in parenthesis*.

**Table 10B:** **Regression of game 2 *ROI* on promise keeping trustees' emotions**.

**Dependent variable**	**Game 2 return/ investment**	**Game 2 return/ investment**	**Game 2 return/ investment**	**Game 2 return/ investment**
**Specification**	**(1)**	**(2)**	**(3)**	**(4)**
Game 1 return-promise	0.611^***^	0.627^***^	0.618^***^	0.681^***^
	(0.225)	(0.226)	(0.225)	(0.223)
Proud	0.070			
	(0.045)			
Believable		0.024		
		(0.050)		
Guilty			−0.208	
			(0.133)	
Ashamed				−0.420^**^
				(0.169)
Constant	0.232	0.365	0.701	0.797^*^
	(0.433)	(0.444)	(0.445)	(0.432)
*R*-squared	0.064	0.050	0.064	0.086
Observations	155	155	155	155

* *Significant at 10%*.

** *Significant at 5%*.

*** *Significant at 1%*.

*Standard errors in parenthesis*.

**Result 5:** Trustees' emotions predict their subsequent demonstrations of trustworthiness such as *signal value* and *ROI* in game 2.

## Discussion

Emotional experiences reported by our participants explain as much as 30.1% of their subsequent behavior. That the studied emotions did not predict more of the observed variance in messaging or trust re-extension may be a consequence of the unexplained variance in reported experiences of emotions. Our model based on game 1 antecedents explained between 20 and 47% of the variance in reports of each of the 12 emotional states studied, with more variance explained for the negative emotion states that were generally experienced with lower intensity. Below we consider whether some of the variance in emotional reports might be explained by differing interpretations of the emotion labels, design limitations of the survey instrument, or deliberately compromised reporting fidelity.

People who are asked to rate single emotions may not be able to accurately describe their emotional states (Ellsworth and Tong, 2006) if emotion experiences are more often and accurately described with multiple words (Izard, 1977), or with different words among different people. While we acknowledge that language could present problems for this research and have no controls, the success of previous research on self-reported emotions in conjunction with experimental games (Ketelaar and Au, 2003; Hopfensitz and Reuben, 2009) gave us encouragement in using our instrument to pursue measures of self-reported emotions following an economic game.

Data quality could also have been affected if our stimulus primed participants to experience specific emotions (e.g., as a consequence of experimenter demand) or if they were incentivized to make untruthful reports. Demand effects to provide inflated reports of the emotional states specifically studied in this report is unlikely because we surveyed a larger set of 20 emotional states and did not reveal the subset of emotional states that we were particularly interested in analyzing. Another concern is that if participants did not view the emotion survey as “incentive compatible,” they may have been motivated to answer untruthfully. A meta-review by Camerer and Hogarth (1999) concludes that there is no clear evidence that additional financial incentives would improve the quality of responses in a simple survey task like ours. In fact, for short tasks like these surveys that people are known to voluntarily complete without problem (because they have sufficient intrinsic motivation to do so), an attempt at increasing participation via financial incentives often “backfires” with counter-intentional effects (e.g., Mellstrom and Johannesson, 2008). Nevertheless, wary of the possibility that participants may have been incentivized to use efficiency tactics to expediently complete the survey (such as by quickly marking all responses the same), we reviewed our data and found no cases of such behavior.

## Conclusion

In this study we examined how participants who were given no indication of opportunity for subsequent interactions, experienced emotions after participating in a trust game, and how such emotions influenced subsequent behaviors when another opportunity for trust-based exchange unexpectedly arose. We found that emotions triggered by trust-based interaction outcomes are predictable and also predict subsequent messaging, apology, trust re-extension, and demonstrated trustworthiness. These findings advance our understanding of human behavior and they contribute to several areas of research.

First, our study provides support for the recalibrational theory of emotions. According to this theory, new information about outcomes triggers emotions, and, when experienced, these emotions recalibrate the system regulating one's propensity for subsequent behavior. In support of this theory, we find that positive emotions experienced after successful trust-based interaction motivate the investor to trust the trustee's cheap signals and re-extend trust, and motivate the *beneficent, promise keeper* trustees to issue shorter re-affirmative messages (word count *M* = 6.33, *SD* = 10.970)^21^ and demonstrate more trustworthiness. Alternatively, a trustee's negative emotional reaction to acting untrustworthy (breaking a promise and exploiting the investor), motivates remedial efforts like longer messages (word count *M* = 19.06, *SD* = 19.031) and apology targeting the affected investor.

Second, our study provides an explanation for why “cheap-talk” (i.e., communication not necessarily affecting incentives, Farrell and Rabin, 1996) is produced despite normative prediction, and why these kinds of messages are often effective. Though cheap-to-produce signals are not guaranteed to be reliable on their own and thus unexpected to persuade receivers (Grafen, 1990; Zahavi, 1993), they are frequently used by humans in the form of spoken or written words to negotiate trust between individuals with conflicting interests (Lachmann et al., 2001). Our study suggests that cheap-to-produce messages are reliable because they are influence by predictable emotions in reliable ways. For example, we find that the predicted positive emotional responses by trustees decrease the likelihood of apology and the length of message. On the other hand, predicted negative emotional responses increase the likelihood of apology and the length of message. Not only do emotions predict the use of cheap-to-produce re-affirmative and remedial messages, but they also predict the subsequent behavior of trustees. In principal, investors who anticipate or have access to the dynamics of these emotional responses and communicative intentions should be able to reliably predict the behavior of trustees based on their messages.

Finally, our study shows that remedial behaviors (spontaneous messaging with apology) can facilitate the rebuilding of damaged trust, with emotions guiding behavioral propensities. This is an important finding, given that breaches of trust are a common problem in social and economic relationships, and corporate life (Robinson and Rousseau, 1994; Barnett, 2003).

For the past couple millennia scholars have recognized that emotions indeed matter in our everyday lives, but have argued over issues of whether and how emotions guide behavior. Much progress has been made toward understanding how emotions are triggered, and what their effects are on behavior. Our study contributes to this discussion by providing evidence that trust-based interaction outcomes trigger emotions in predictable ways that, in turn, influence our propensity toward subsequent behaviors. One drawback of our study is that we elicited emotions using self-reports. Future research can overcome this limitation by focusing on objective neurological, physiological, and behavioral measures of emotional states.

## Author contributions

For inspiration to pursue this study, we thank John Dickhaut. For helpful comments, we thank the associate editor, three anonymous referees, Hilly Kaplan, Greg Bryant, and participants at the Workshop on Communication in Games (at the University of Zurich), the Human Behavior and Evolution Society annual meeting (in Montpellier, France), the Center for Evolutionary Psychology (at UC Santa Barbara), the John Dickhaut Memorial Conference (at Chapman University), and the Association for Religion, Economics and Culture annual meeting (at Chapman University). We would also like to thank the Economic Science Institute at Chapman University for funding this research.

### Conflict of interest statement

The authors declare that the research was conducted in the absence of any commercial or financial relationships that could be construed as a potential conflict of interest.

## Footnotes

^1^A number of other behavioral economic studies have also used veiled designs where interacting participants are unaware of opportunity for repeated interaction(s) that will later be made available (e.g., see Binmore et al., 1985; Burnham et al., 2000; Ellingsen et al., 2010; Gambetta and Székely, 2014). Ellingsen et al. (2010, p. 96) discuss why the veiled design avoiding deception by commission does not violate the non-deception norm in behavioral economic experiments and why withholding procedural information may serve to limit undesirable experimenter demand. Nevertheless, because there is no clear agreement among economists as to what kinds of deception are taboo (see survey results by Krawczyk, 2013 and discussion by Wilson, 2014), deception by omission remains a potential concern with our design. Veiled designs may create negative externality in that the next time participants return to the laboratory to participate in other studies, they may question whether they should anticipate unannounced tasks or interactions, unless the instructions explicitly exclude such a possibility (see also Davis and Holt, 1993, pp. 23–24; Wilson, 2014). It is unlikely that our design produced these externalities, however, as we followed the procedural norm for conducting research with our participant pool by specifying the expected session duration, making a clear statement about the experimental game which was the final one in the session, and indicating when payments would be issued. We discuss in the design section how future studies using veiled designs can make improvements over ours to avoid omitting important details—namely, whether subsequent tasks are scheduled as part of the session.

^2^While transactions with strangers may have been full of danger, mistrust, and exploitation for much of human evolutionary history (Diamond, 1997; Bowles, 1998), at least more recently, since the 19th century, as modern market society and per capita income have grown (Clark, 2008), the norm of exchange has moved from more exclusively personal to more anonymous (North, 1990). Market proliferation, in turn, has reinforced learning of the notion that trust in strangers brings benefits, resulting in greater trustworthiness (Henrich et al., 2001, 2010; Zak and Knack, 2001; Al-Ubaydli et al., 2013).

^3^McNally and Tanner (2011) speculated that under conditions of “an unforgiving Machiavellian society,” one-shot cooperation is most likely to evolve.

^4^As suggested by the phrase “… fool me twice, shame on me,” there may be stronger hedonic costs to being a targeted victim of repeated deception, than are experienced after being a first-time victim.

^5^In this study we focus on 12 emotions that had previously been shown to be affected by trust-based exchange outcomes (Schniter and Shields, 2013; Schniter et al., 2014). However, to avoid demand effects that might result by focusing participants only on the 12 emotions of interest to our study, and to limit post-rationalization that might result from inducing a more limited focus, we presented subjects with a larger number of 20 emotions. This set of 20 emotions is frequently used in versions of the one-dimensional Positive and Negative Affect Scale (PANAS), a self-report measure of positively and negatively valenced affect state activations developed by Watson et al. (1988) that has been demonstrated across large non-clinical samples to be a reliable and valid measure of these states (Crawford and Henry, 2004).

^6^Human psychological adaptations for sociality likely evolved under a selective regime characterized by repeated interactions among known others (Kelly, 1995).

^7^Messaging is often an option (i.e., not compulsory). When optional, we expect people to tradeoff costs and benefits. Messaging may *not* be chosen for a variety of reasons including: to minimize cognitive effort, in an attempt to manage impressions, to save time, to pursue alternative opportunities that otherwise might be forgone.

^8^Despite being difficult to control, people regularly make efforts to control their experience of emotions and the effects of those emotions on decision making processes (Gross, 2002). Indeed, it has been shown that an individual's ability to effectively control their emotions has implications for their decision making processes under uncertain conditions (Heilman et al., 2010). While individual differences in *degree* of emotional regulation likely explain some variance in the behaviors that we explore with this study, our recalibrational model is not a model of individual differences. Rather, we study the different *kinds* of emotional adaptations that should have reliably predictable directional effects among all people.

^9^Schniter et al. (2014) also collected data on emotional reactions to trust-based interaction outcomes using an emotional status survey with multiple emotion items. They reported floor effects with some of their items: where participants indicated experiencing the emotion very little or not at all. However, other emotions in their study showed strong responsiveness to trust-game outcomes, and based on those results they suggested that future researchers consider investigating appreciation, happiness, pride, frustration, anger, and guilt: a selected set with balanced valance that is representative of the varieties of functions described by their recalibration model of emotions in trust-based interaction. We chose to study those six emotions in addition to another six which we expected to be roughly synonymous and therefore similarly activated: cheerfulness (often concomitant with appreciation), contentment (often concomitant with happiness), believability (often concomitant with pride), aggravation (often concomitant with frustration), disgust (often concomitant with anger), and shame (concomitant with guilt when the offender's culpability is known).

^10^A few neuroeconomic studies have shown evidence of neural correlates of interaction behaviors in ultimatum games (Sanfey et al., 2003) and trust games (Aimone et al., 2014) that may correspond to emotional experiences (e.g., see Takahashi et al., 2004) but have not provided direct measures of whether emotions were actually experienced. Because physiological measures are often incapable of detecting social emotions (Adolphs, 2002), self-reports may provide the best, if not only, direct measures of social emotions (Hopfensitz and Reuben, 2009).

^11^Though we failed to provide information at the outset of our session indicating our intent to provide a repeated trust game opportunity, we provided participants a reasonable indication of expected total duration for the session to which they were recruited, and we made no misleading or counterfactual statements. As such, our procedure does not qualify as the more typical form of experimental deception (i.e., *by commission* of lies or exaggerations). Nevertheless, future studies using similar veiled approaches can improve on our design by more explicitly clarifying from the outset that a multi-stage approach is planned. As suggested by the editor, a veiled design could avoid use of the word “experiment” in describing a single stage and more transparently explain to participants something like, “The session consists of three stages. Now you will receive the instructions of stage 1. After completion of stage 1, there will be a stage 2 for which you will receive a new set of instructions. Finally, following completion of stage 2 there will be a final stage 3 for which you will receive a new set of instructions.”

^12^We were motivated to see how people deal with trust re-extension dilemmas that were not explicitly anticipated as a consequence of certain expectations (for repeated interaction) but arise through a surprise unveiling. This required us to first examine trust-based interaction behaviors carried out by procedurally naïve individuals from behind the veil of ignorance.

^13^Of the 20 emotions surveyed, we later focus predictions and analyses on a positive subset (appreciation, contentment, cheerfulness, happiness) and a negative subset (anger, disgust, aggravation, frustration) among investors (who might have been benefited or exploited and had their trust assured or damaged), and a positive subset (pride, believability) and a negative subset (guilt, shame) among trustees (who might have been beneficent or opportunist and kept or broken their promises).

^14^The instructions for coders, details about how they were paid, and their earning from the incentivized task are reported in Schniter et al. (2013).

^15^We use a standard approach from content analysis methodology to calculate Cohen's Kappa inter-rater agreement coefficient (Cohen, 1960; Krippendorff, 2004). Kappa values between 0.41 and 0.60 are considered “Moderate” agreement, and those above 0.60 indicate “Substantial” agreement (Landis and Koch, 1977).

^16^These separate effects might come about as a result of reactions to two distinct phenomena: one being the abuse of language (e.g., issuing a deceptive promise), and the second being the imposition of economic harm (returning less than invested). These two phenomenon are not necessarily related: deceptive promises can be made by trustees providing positive *ROIs* (observed in our sample), just as it is possible for promise keeping trustees to provide negative *ROIs* (unobserved in our sample).

^17^We suspect that investor emotional reactions to trustee re-affirmative and remedial behaviors would also affect our model, however we did not survey emotional reactions to either received messages or game 2 promises and are therefore unable to account for their partial effects. Our model characterizes cooperation problems resulting from certain game interaction outcomes as discrete and binary (e.g., a promise is either *kept* or *broken*), and expects the set of recalibrational emotions they trigger to be distinct (promoting either *re-assuring* or *remedial* messaging, respectively). As such, we evaluate reports of emotional states and behavioral differences between naturally-formed groups (e.g., *promise keeper* vs. *promise breaker* trustees, *beneficent* vs. *opportunist* trustees, etc.) using two-sample (Welch's) t-tests for equal means where appropriate.

^18^Moderately high reliability of internal consistency in PANAS similar to ours has been reported by Watson et al. (1988) and others (e.g., Mehrabian, 1998; Roesch, 1998; Kausel and Connolly, 2014).

^19^ We refer to reports of proud, believable, guilty, ashamed, angry, disgusted, aggravated, frustrated, appreciative, content, cheerful, and happy emotional states.

^20^*Message, word count*, and *apology* are inter-related: whether or not there is a *message* (with content) is related to *word count*; whether or not there is *apology* is related to word count and to *message*.

^21^Space precludes a full content analysis, but cursory inspection reveals that these messages tend to be re-affirmative—calling attention to the successful exchange and intention to repeat it. Examples include “teamwork!,” “same deal.,” “same as last time:),” “we're a good pair. I don't know what else to say haha.,” “same thing?,” “lets do this!,” “Pleasure doing business with you,” “I will keep it equal like last time,” “Let's just do the same transfer again,” “Let's do the same… It worked and we both made some money!!!,” “Same deal as before sounds about right, in my opinion,” “Let's do the same thing, that way we both get the max amount of money,” “Same thing again. We both benefit.,” “I like the way we did it last time, it works out nicely for both of us and it's fair:) Thanks for being great!,” “Heyo- happy to work with you again ¶ and do the same thing.,” “well we worked together so far- want to do it again? at least we'll both make more than $5.” Interested readers are encouraged to further examine the message content in Supplementary Material.
