# Supplementary material for: Predictable and predictive emotions: explaining cheap signals and trust re-extension
Source: Front Behav Neurosci. 2014 Nov 20;8:401. doi: 10.3389/fnbeh.2014.00401 (PMC4238347; doi:10.3389/fnbeh.2014.00401)
Supplement: Supplementary file 1 [file Presentation1.PDF]

Appendix A: Instructions

INSTRUCTIONS

Thank you for participating in this experiment. The purpose of this experiment is to study how people make decisions in a particular situation. Feel free to ask us questions as they arise, by raising your hand. Please do not speak to other participants during the experiment. You will receive $7 for participating in this session. You may also receive additional money, depending on the decisions made (as described below). Upon completion of the session, this additional amount will be paid to you individually and privately.

During the session, you will be paired with another person. However, no participant will ever know the identity of the person with whom he or she is paired.

DECISION TASKS

In each pair, one person will have the role of A, and the other will have the role of B. The amount of money you earn depends on the decisions made in your pair.

First, by choosing a dollar amount from $0 to $20, B indicates the proportion of a possible $20 income that he or she promises to transfer back to A, should A choose IN. Specifically, B will complete the following statement: "I (Participant B) promise to transfer back ___ of my income to you (Participant A) if you choose IN". The computer will convey B's statement to A, and then A and B will proceed as described below. B may still choose an amount to transfer back to A that is different than the amount promised.

Having received a statement from B, A indicates whether he or she chooses IN or OUT. If A chooses OUT, A receives $5 and B receives $0. If A chooses IN, then B receives $20 income. In such a case, after receiving $20 income, B must choose a dollar amount from $0 to $20 to transfer back to A.

SURVEY

After having completed the decision tasks described above you will be asked to fill out a short 20 item survey.

DIAGRAM

The following diagram represents how the experiment proceeds:

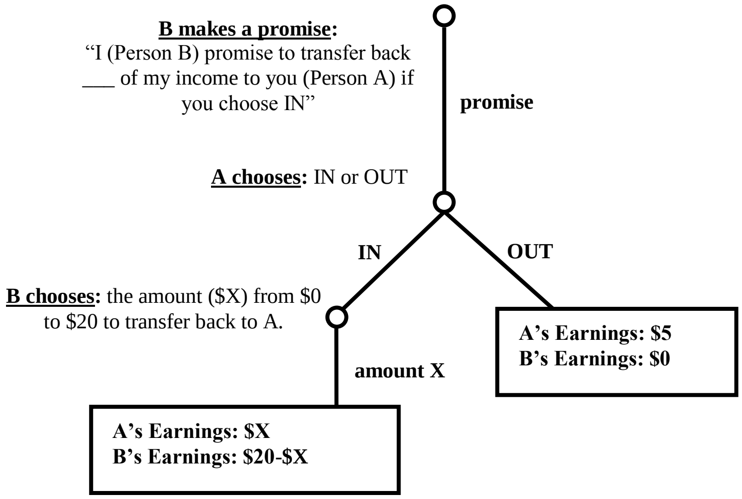

(This part of the instructions was handed out after the first "experiment" was conducted.)

REPETITION OF THE EXPERIMENT

The same decision tasks that were just completed will be repeated again, with everyone remaining in the same A or B roles and paired with the same participants as in the previous tasks.

MESSAGE

Prior to repetition of the previous decision tasks, B has an option to send a message to A. B may use a text box to type a message, if desired. We will allow time as needed to construct and type messages. When B's message has been completed (by typing in the text box and clicking on the send button) it will be conveyed by the computer to the appropriate Participant A, and then A and B will proceed with decision tasks. In these messages, no one is allowed to identify him or herself by name, number, gender, or appearance. Other than these restrictions, B may say anything in the message. If you wish not to send a message, simply click on the send button without having typed anything in the message box.

DECISION TASKS AND SURVEY (REPEATED AS BEFORE)

This second set of decision tasks and the accompanying 20 item survey is the final part of the experiment. There will be no further tasks.

Appendix B: 20-item Emotion Survey

The following scale consists of a number of words that describe different feelings and emotions. Read each item and then mark the appropriate answer in the space next to that word. Indicate to what extent the experiment in which you just participated made you feel.

Use the following scale to record your answers:
(1) very slightly or not at all, (2) a little, (3) moderately, (4) quite a bit, (5) extremely

Guilty	1 ○ ○ ○ ○ ○ 5	Secure	1 ○ ○ ○ ○ ○ 5
Embarrassed	1 ○ ○ ○ ○ ○ 5	Angry	1 ○ ○ ○ ○ ○ 5
Proud	1 ○ ○ ○ ○ ○ 5	Disgusted	1 ○ ○ ○ ○ ○ 5
Ashamed	1 ○ ○ ○ ○ ○ 5	Jealous	1 ○ ○ ○ ○ ○ 5
Inspired	1 ○ ○ ○ ○ ○ 5	Surprised	1 ○ ○ ○ ○ ○ 5
Depressed	1 ○ ○ ○ ○ ○ 5	Appreciative	1 ○ ○ ○ ○ ○ 5
Believable	1 ○ ○ ○ ○ ○ 5	Cheerful	1 ○ ○ ○ ○ ○ 5
Content	1 ○ ○ ○ ○ ○ 5	Aggravated	1 ○ ○ ○ ○ ○ 5
Happy	1 ○ ○ ○ ○ ○ 5	Frustrated	1 ○ ○ ○ ○ ○ 5
Triumphant	1 ○ ○ ○ ○ ○ 5	Sad	1 ○ ○ ○ ○ ○ 5

OK

Appendix C: Comparisons of Emotions between Trustees and between Investors

Here we provide bar charts of trustees' and investors' emotions and report the details of Welch's t-test comparisons between different groups of trustees (i.e., *promise breaker* vs. *promise keeper, opportunist* vs. *beneficent*) and investors (i.e., *damaged* vs. *assured, exploited* vs. *benefited*). These groups are hypothesized to have encountered distinct cooperation problems resulting from certain game interaction outcomes and we classify them accordingly. Trustees are classified into the following groups: *promise breakers* when game 1 return – game 1 promise < 0, *promise keepers* when game 1 return – game 1 promise ≥ 0, *opportunists* when game 1 return / game 1 investment ≤ 1, and *beneficent* when game 1 return / game 1 investment > 1. Investors are classified into the following groups: those with *damaged trust* when game 1 return – game 1 promise < 0, those with *assured trust* when game 1 return – game 1 promise ≥ 0, those who were *exploited* when game 1 return / game 1 investment ≤ 1, and those who *benefited* when game 1 return / game 1 investment > 1.

Figure C1: Bar graphs *Promise Breaker* and *Promise Keeper* Trustee Emotions

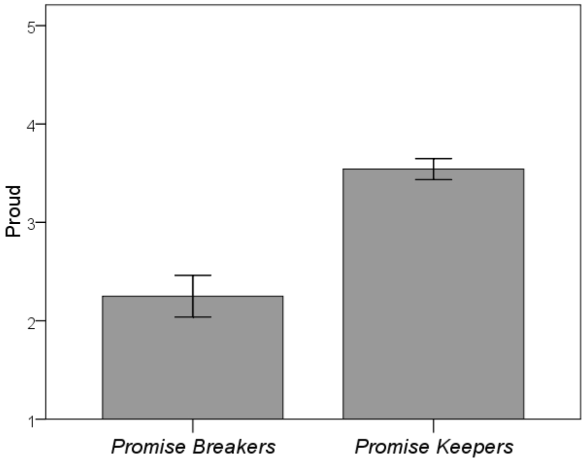

The difference was significant ($t(189) = -5.291$, $p < .001$) between *promise keepers'* M = 3.54 (SD = 1.330) and *promise breakers'* M = 2.25 (SD = 1.273) reports of pride.

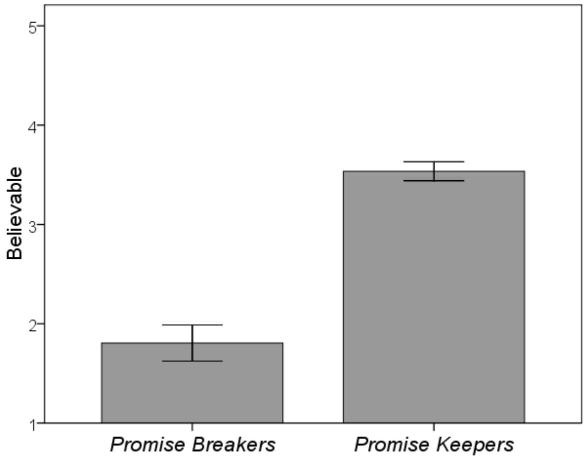

The difference was significant ($t(189) = -7.969$, $p < .001$) between *promise keepers'* M = 3.54 (SD = 1.191) and *promise breakers'* M = 1.81 (SD = 1.091) reports of believability.

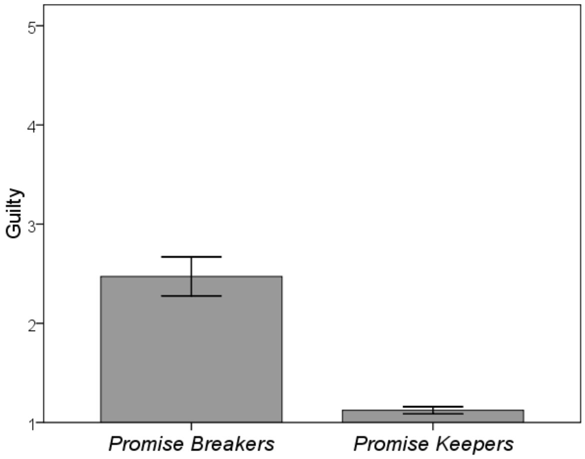

The difference was significant ($t(189) = -11.238$, $p < .001$) between *promise keepers'* M = 1.12 (SD = 0.446) and *promise breakers'* M = 2.47 (SD = 1.183) reports of guilt.

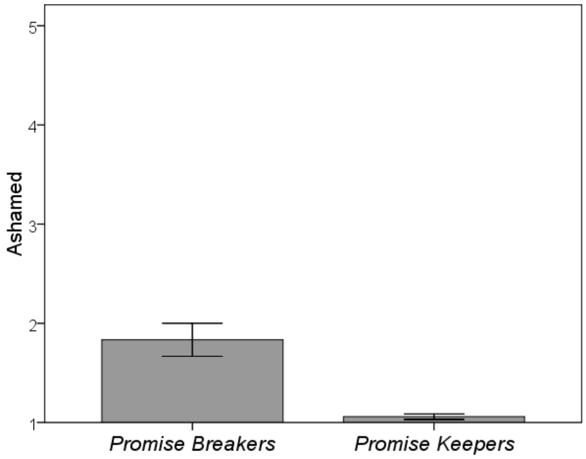

The difference was significant ($t(189) = -7.878$, $p < .001$) between *promise keepers'* M = 1.06 (SD = 0.346) and *promise breakers'* M = 1.83 (SD = 1.000) reports of shame.

Figure C2: Bar graphs *Opportunist* and *Beneficent* Trustee Emotions

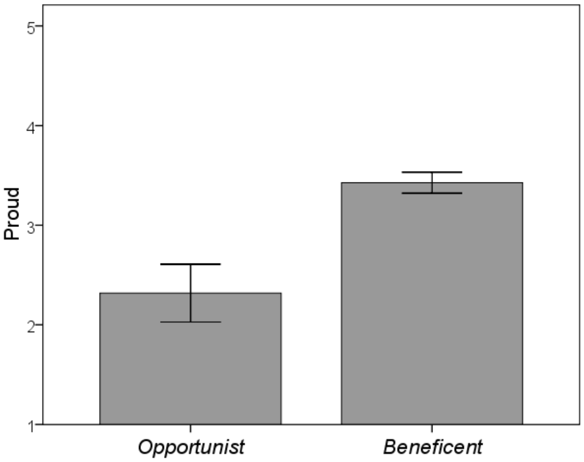

The difference was significant ($t(189) = -6.965$, $p < .001$) between *beneficent* M = 3.43 (SD = 1.370) and *opportunist* trustees' M = 2.00 (SD = 1.340) reports of pride.

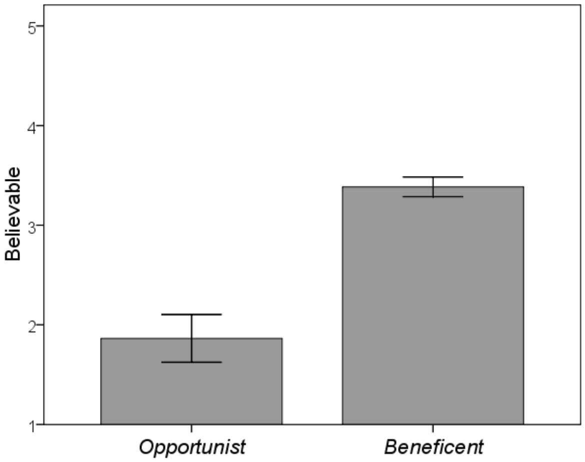

The difference was significant ($t(189) = -6.203$, $p < .001$) between *beneficent* M = 3.38 (SD = 1.282) and *opportunist* trustees' M = 2.18 (SD = 1.308) reports of believability.

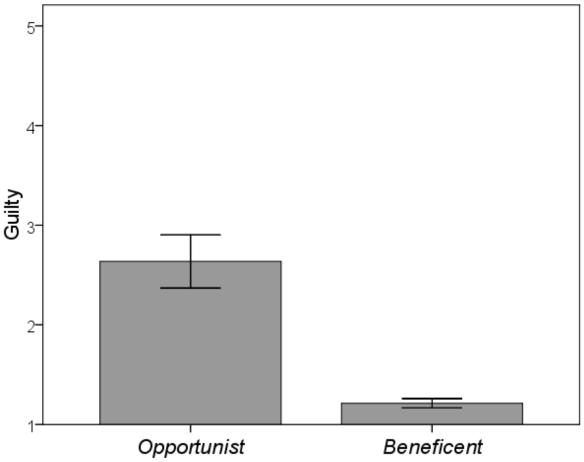

The difference was significant ($t(189) = 4.316$, $p < .001$) between *beneficent* M = 1.21 (SD = 0.599) and *opportunist* trustees' M =1.73 (SD = 1.205) reports of guilt.

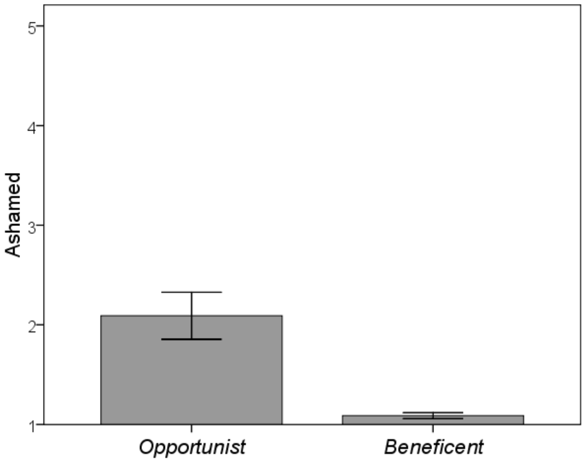

The difference was significant ($t(189) = 5.833$, $p < .001$) between *beneficent* M = 1.09 (SD = 0.391) and *opportunist* trustees' M =1.63 (SD = 1.025) reports of shame.

Figure C3: Bar graphs of Emotions for Investors who had Trust *Damaged* and *Assured*

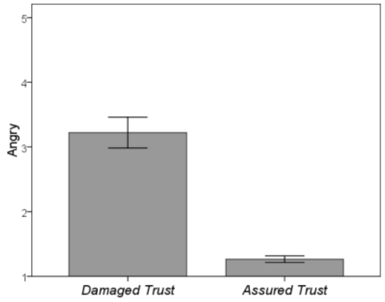

The difference was significant ($t(189) = -12.644$, $p < .001$) in reports of anger between *assured trust* M = 1.26 (SD = 0.625) and *damaged trust* M = 3.22 (SD = 1.436) investors.

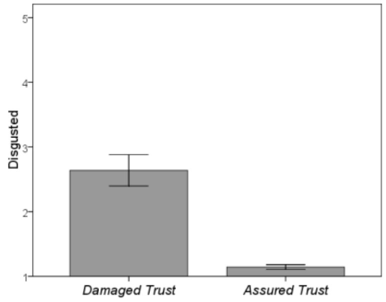

The difference was significant ($t(189) = -10.645$, $p < .001$) in reports of disgust between *assured trust* M = 1.14 (SD = 0.476) and *damaged trust* M = 2.64 (SD = 1.457) investors.

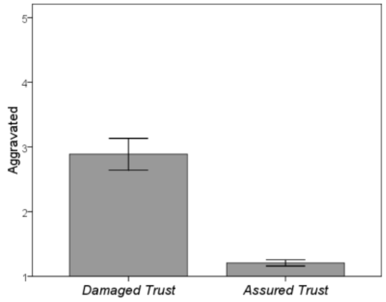

The difference was significant ($t(189) = -11.009$, $p < .001$) in reports of aggravation between *assured trust* M = 1.21 (SD = 0.589) and *damaged trust* M = 2.89 (SD = 1.469) investors.

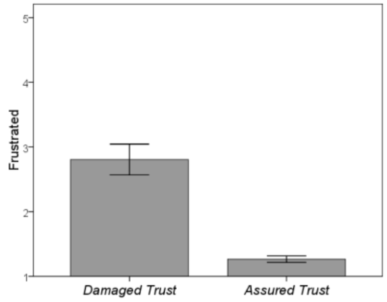

The difference was significant ($t(189) = -10.051$, $p < .001$) in reports of frustration between *assured trust* M = 1.26 (SD = 0.615) and *damaged trust* M = 2.81 (SD = 1.431) investors.

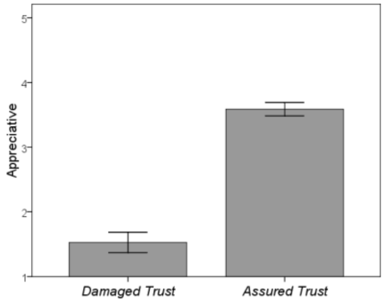

The difference was significant ($t(189) = 9.071$, $p < .001$) in reports of appreciation between *assured trust* M = 3.59 (SD = 1.283) and *damaged trust* M = 1.53 (SD = 0.941) investors.

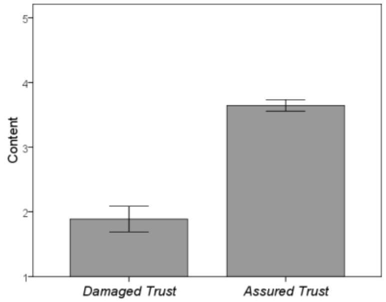

The difference was significant ($t(189) = 8.476$, $p < .001$) in reports of contentment between *assured trust* M =3.65 (SD = 1.103) and *damaged trust* M =1.89 (SD = 1.190) investors.

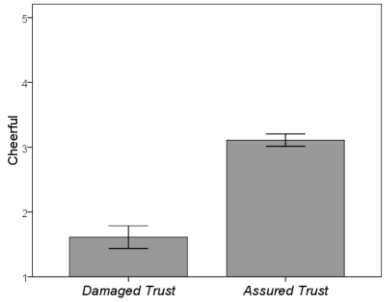

The difference was significant ($t(189) = 6.937$, $p < .001$) in reports of cheerfulness between *assured trust* M =3.11 (SD = 1.193) and *damaged trust* M = 1.61 (SD = 1.050) investors.

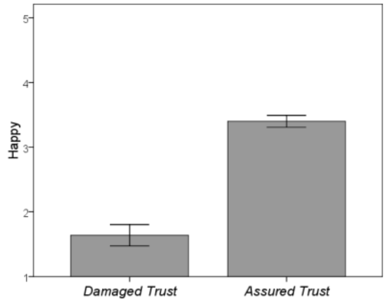

The difference was significant ($t(189) = 8.491$; $p < .001$) in reports of happiness between *assured trust* M =3.40 (SD = 1.149) and *damaged trust* M =1.64 (SD = 0.990) investors.

Figure C4: Bar graphs of Emotions for *Exploited* and *Benefited* Investors

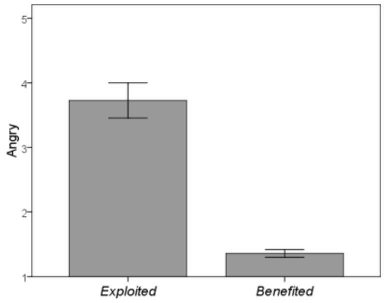

The difference was significant ($t(189) = 12.337$, $p < .001$) in reports of anger between *benefited* M = 1.36 (SD = 0.775) and *exploited* M = 3.73 (SD = 1.279) investors.

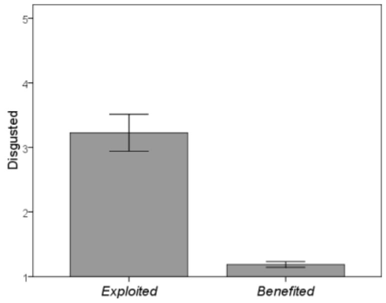

The difference was significant ($t(189) = 12.763$, $p < .001$) in reports of disgust between *benefited* M = 1.193 (SD = 0.577) and *exploited* M = 3.23 (SD = 1.343) investors.

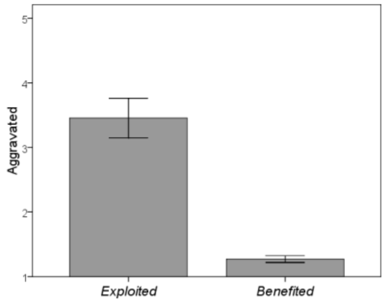

The difference was significant ($t(189) = 12.138$, $p < .001$) in reports of aggravation between *benefited* M = 1.27 (SD = 0.67) and *exploited* M = 3.45 (SD = 1.438) investors.

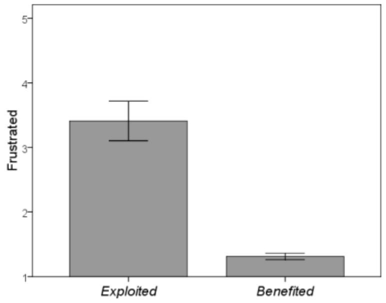

The difference was significant ($t(189) = 11.920$, $p < .001$) in reports of frustration between *benefited* M = 1.31 (SD = 0.647) and *exploited* M = 3.41 (SD = 1.436) investors.

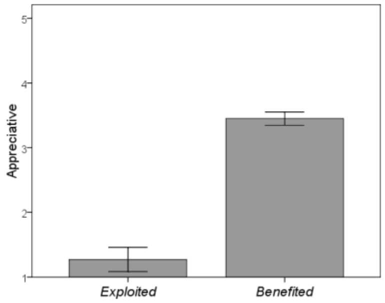

The difference was significant ($t(189) = -7.426$, $p < .001$) in reports of appreciation between *benefited* M =3.45 (SD = 1.336) and *exploited* M = 1.27 (SD = 0.883) investors.

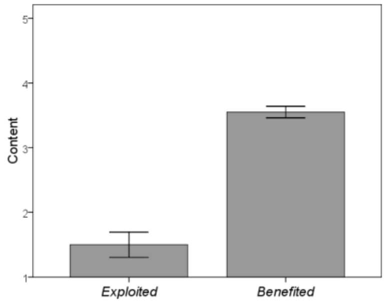

The difference was significant ($t(189) = -7.940$, $p < .001$) in reports of contentment between *benefited* M = 3.55 (SD = 1.165) and *exploited* M = 1.50 (SD = 0.913) investors.

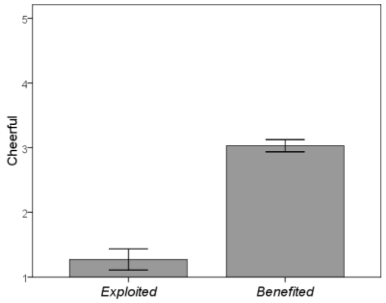

The difference was significant ($t(189) = -6.568$, $p < .001$) in reports of cheerfulness between *benefited* M = 3.03 (SD = 1.222) and *exploited* M = 1.27 (SD = 0.767) investors.

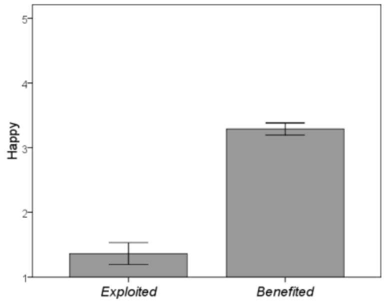

The difference was significant ($t(189) = -7.304$, $p < .001$) in reports of happiness between *benefited* M = 3.29 (SD = 1.202) and *exploited* M = 1.36 (SD = 0.790) investors.

Appendix D: Promises and Messages

Table D1: Promise-Breakers Messages

Promised Game 1	Returned Game 1	Message	Word-count	Broad Apology	Promised Game 2	Trusted? Game 2	Returned Game 2
15	0	Let's split even. $10 and $10.	6	YES	10	YES	8
10	1	If I knew there were 2 rounds I would have split it up even the first round. This round I'll make it up to you by giving you 15 if you're IN, this way we both end up with more money. Sorry again.	43	YES	15	YES	3
10	0	Hey im sorry about that I didn't realize there was going to be another round.! Let me make things right.	20	YES	15	YES	5
10	9	i'll do the same deal as last time, sound fair?	10	NO	15	YES	9
10	7	to even out i will give you 13 and i will take 7	13	YES	13	YES	10
10	0	doooooooood we all here to make muney baby so why dont we just split this huney down da middle, a lil lovin for da both of us? ill forrealze give you like 10 bucks and ill keep 10 you dig? stay fresh ;)	43	YES	10	YES	0
10	7	How much would you want this time seeing how you didn't have a choice last time? I'm willing to make it even between the two of us.	27	YES	13	YES	0
10	5	Hello A! I'm sorry I fell back on my promise, haha. To be honest, I'm dead broke and I haven't eaten all day and I'm literally about to run out of gas in my car, and those extra five dollars are going to help me out with that! ¶ If you choose out the most you are going to get is 5 more dollars, I can promise you that I'll agree to give you $10 if you choose in. Hopefully this works out! Either way, have a good one!	88	YES	12	YES	12
8	5	I only sent less than promised because I wanted to see what would happen	14	YES	10	YES	10
10	2	Hi, I was a little confused as to the experiment before. But I will stay true to this promise	19	YES	9	YES	9
13	9	lets split the money 10 and 10	7	YES	10	YES	10
10	8	I apologize for cheating you out of your $2 - normally I'm not the kind of person to do that sort of thing. When two people aren't face-to-face they usually have more confidence to do things they wouldn't normally do. This time I promise I'll play fair.	47	YES	9	YES	9
10	7	I didn't know we were repeating this. This time I really will split 50/50 :)	15	YES	10	YES	10
11	2	I will transfer back 18 to you this time to make it fair ¶ so we will have the same amount. I promise this time.	24	YES	18	YES	10
14	11	Hi. I apologize for short changing you. I should have been honsest and gone off the first example. I went off the third example w/my self interest in mind. I'll keep my word this time.	35	YES	18	YES	18
11	10	10/10?	1	YES	12	YES	7
8	0	sorry about last time i feel bad......50/50 this time?	10	YES	10	YES	0
8	7	Strategy :)	1	YES	10	YES	10
10	0	I feel bad that you now only have the option of going home with $5 so you should click in again and i will give you $12 so that you go home with more than just the basic amount possible	40	YES	12	YES	0
8	6	My sincerest appologies on that last one...I do feel quite guilty ¶ and I assure you that this time I shall keep my promise with utmost integrity. You have	34	YES	16	YES	1

		my word as an honest gentleman.						
**10**	**7**	I feel bad for promising 10 and giving you 7. If you choose in I'll send you 13 so that we'll come out even.	**24**	**YES**	**13**	**YES**	**13**
**5**	**1**	my bad...	**2**	**YES**	**10**	**YES**	**8**
**10**	**3**	I'll transfer back more money this time ¶ actually $10	**9**	**YES**	**11**	**YES**	**10**
**10**	**8**	Hi ¶ I was testing if it really will let me decide how much I can get myself. This time I will give you the right amount I promised.	**28**	**YES**	**10**	**YES**	**10**
**10**	**0**		**0**	**NO**	**20**	**YES**	**0**
**10**	**0**	This time I'll give you what I promise. Sorry!	**9**	**YES**	**10**	**NO**	
**10**	**0**	ok for real this time haha. The first time was a joke lol	**13**	**YES**	**15**	**NO**	
**17**	**0**	May God bless you	**4**	**NO**	**13**	**NO**	
**10**	**7**	In the previous exercise I wanted to see if one really could promise one amount and then give another. After seeing that it is possible, I promise to give you the amount I state.	**34**	**YES**	**10**	**NO**	
**17**	**1**	i know that there is no reason you'd trust me because i didn't follow through with my promise last time ¶ but if you choose in i will transfer all of the money that i say i will. ¶ for real this time.	**41**	**YES**	**18**	**NO**	
**10**	**5**	Even though I was decietful ¶ you were no worse off then had you picked OUT. The other option would have still led you to $5.	**25**	**YES**	**5**	**NO**	
**12**	**5**		**0**	**NO**	**20**	**NO**	
**18**	**4**		**0**	**NO**	**12**	**NO**	
**7**	**6**		**0**	**NO**	**9**	**NO**	
**10**	**5**		**0**	**NO**	**8**	**NO**	
**10**	**7**		**0**	**NO**	**10**	**NO**	

Table D2: Distrusted Trustees Messages

Promised Game 1	Message	Word-count	Promised Game 2	Trusted? Game 2	Returned Game 2
**11**	8 seems fair	**3**	**8**	**YES**	**0**
**16**	lets go 50/50. i give you $10, i get $10. ¶ its almost christmas....	**13**	**10**	**YES**	**1**
**9**	I want to split the money right down the middle. I will give you ten dollars and I will get ten dollars. If you choose out you will get less and both of us will come out empty handed. This is for the benefit of both parties and you will make more money in this way than you will by opting out.	**62**	**10**	**YES**	**1**
**5**	hey if i transfer 9 to you will you accept ?	**11**	**9**	**YES**	**8**
**10**	Trust me this time. Please?	**5**	**10**	**YES**	**7**
**10**	hi! i was actually going to give you the $10 that time! You would have made more money! I promise to give what I promise to you this time as well!	**31**	**8**	**YES**	**5**
**11**	Hello, ¶ I think $11.00 for you is a fair price for this survey and it is more than the $5.00 you get for choosing Out. I will keep my offer the same if you chose In. ¶ Thank you	**37**	**11**	**YES**	**12**
**10**	Hey! Okay, listen, I was genuinely going to give you ten dollars. I think it makes sense for both of us to make as much money as possible. I'm not trying to trick you. I'm just poor and want a few extra dollars to buy Christmas presents. So could you please just be in" next time? That way we can both make more. I promise I am not lying to you. I know it's anonymous but please trust me. :("	**80**	**9**	**YES**	**10**
**6**	You click out, you earn 5. You accept my offer, you earn 6. It doesn't make any sense to click OUT. This is not a situation where my gain affects your profits in the future, this isn't one business earning a little bit and another earning a lot at its expense. You have to option of $6 or $5, without repercussions or any damage in the future. Me getting 0 does you no good, all it does is hurt you. If you want $5, click OUT. But it obviously makes more sense to click IN.	**95**	**6**	**YES**	**7**
**1**	If I offer you at least 30% of my income we both make more than if you opt out.	**19**	**7**	**YES**	**7**
**8**	How about 10? We will both make the same amount evenly.	**11**	**10**	**YES**	**10**
**10**	I am a person of my word. I will transfer back $10 so we both make the same amount of money and more money than if you pick OUT	**29**	**10**	**YES**	**10**
**10**	I will offer 10 dollars of my income to you. If you choose in, then you will recieve 10 dollars and i will recieve 10 dollars. If you choose out, you will only recieve 5 dollars.	**36**	**10**	**YES**	**10**
**20**	I will split it with you so we both get ten dollars.	**12**	**10**	**YES**	**10**
**6**		**0**	**9**	**YES**	**9**
**6**	Please trust me when I say I will give you the amount I will promise you. This way, we will both earn more money instead of you just earnint $5 and me earning nothing. Let's take all of their money together!	**41**	**7**	**YES**	**7**
**5**	I will transfer 10 dollars.	**5**	**10**	**YES**	**10**
**5**	Hey ¶ to make this a win-win situation for both of us ¶ I'll transfer $10 and that way both of us will earn the same amount. It's really a good gameplan. :)	**31**	**0**	**YES**	**8**
**7**	Ok ¶ so this time let's make it actually fair.... I should have made it even last time. So this time if I give you back $8 ¶ you'll leave with $20 and I'll leave with $19. you still come out on top ¶ but I don't mind. And that's more than you'll make if you click OUT. I'm in the same boat as you....I too am poor as hell and would like to make some easy cash....	**75**	**8**	**YES**	**8**
**20**	I will transfer you back 75% back.	**7**	**10**	**YES**	**0**
**5**	We can figure out a way to divide the amount of the $20 equally if the result from that will have us leave here with more than $7	**28**	**10**	**YES**	**9**
**10**	I won't ask you to trust me. That's your choice ¶ what I will say though is offer you $10 to each of us. We both walk away from this evenly and both better off than we came in.	**38**	**10**	**YES**	**10**
**4**	I promise to transfer back 20 of my income to you. I really need this extra money. I hope you understand	**21**	**20**	**YES**	**0**
**9**	Let's be fair and split the pool evenly. Trust that I will not go back on what I say.	**19**	**10**	**YES**	**10**
**6**	I will give you half of the amount of the income	**11**	**12**	**YES**	**0**
**5**	Hello A ¶ I'm stoked to be making money while my roomate snores away. Hahaha. ¶ Cha-ching ly ¶ B.¶	**16**	**4**	**YES**	**4**

**20**	if i say $20 and you accpet ¶ I promise to give you $20 back so we both leave with $20 ¶ the max amount	**23**	**20**	**YES**	**20**
**8**	I promise to uphold any deals set before me	**9**	**10**	**YES**	**10**
**6**	I promise to give you $7 for clicking "in." I guarantee it. As I see it ¶ this gives you $2 more dollars than you would recieve by clicking "out." It's a win-win situation.	**33**	**7**	**YES**	**7**
**8**		**0**	**10**	**YES**	**0**
**5**	I think you should choose IN because it is simple game theory. If you choose IN and I choose to give you $10 ¶ which I promise to give you ¶ then we both win. I know that you would automatically want to choose OUT so that you can get $5 no matter what ¶ but I promise you that you will get $10. We both want to get money ¶ and this is a good way to share our earnings. I hope you choose IN! :)	**83**	**5**	**YES**	**0**
**10**	If I offered you 10$ why would you rather get 5?	**11**	**10**	**YES**	**10**
**6**	Ouch.): I'm not gonna scam you, dude. When I make a promise, I make a promise. We both make more money this way; it's good all around!	**28**	**6**	**NO**	
**5**	hey Participant a make a deal dont do like this we should come here to earn money kul	**18**	**1**	**NO**	
**10**	dont be an asshole	**4**	**7**	**NO**	
**10**	Trust me.	**2**	**10**	**NO**	
**6**	You'll get more than $5.	**5**	**8**	**NO**	
**8**	Hello there! So it's probably hard to trust me ¶ in that I will return your money? And I would quite frankly feel the same way. The thing is though that you don't know me but I know me and I know that when I make a promise I keep it. I hope you can trust in me. :)	**58**	**7**	**NO**	

Table D3: Promise-Keepers Messages

Promised	Returned	Message	Word-count	Promised Game 2	Trusted? Game 2	Returned Game 2
**6**	**14**	I paid out more than I promised to transfer back the first time as a reward for going IN	**19**	**10**	**YES**	**0**
**6**	**6**	merry christmas!	**2**	**10**	**YES**	**10**
**10**	**10**	Same deal as before sounds about right, in my opinion.	**10**	**10**	**YES**	**10**
**8**	**8**	i guess you need the money too so we should split it!	**12**	**10**	**YES**	**10**
**10**	**10**	Hey there. Want to do the same thing again, and both come out ahead?	**14**	**10**	**YES**	**10**
**9**	**9**	hey so 10 and 10 this time?	**7**	**10**	**YES**	**10**
**10**	**10**	I will split it equally	**5**	**10**	**YES**	**10**
**10**	**10**	Thanks for accepting my last offer. I promise to always uphold my side of the deal.	**16**	**10**	**YES**	**10**
**9**	**9**	hello A! :)	**3**	**7**	**YES**	**7**
**7**	**7**	I won't lie to you. I know we're all broke college students here who need to make money. ugh	**19**	**8**	**YES**	**8**
**10**	**10**	This is tres bizarre.	**4**	**10**	**YES**	**10**
**5**	**5**	i send you 10 and you hit in..that way we both get the same amount of money. =]	**19**	**10**	**YES**	**10**
**10**	**10**	Let's do the same thing, that way we both get the max amount of money	**15**	**10**	**YES**	**0**
**10**	**10**	we'll go 50/50 on everything. i promise.	**7**	**10**	**YES**	**10**
**6**	**6**	we're a good pair. i dont know what else to say haha.	**12**	**6**	**YES**	**1**
**11**	**11**	expecto patronum!	**2**	**11**	**YES**	**2**
**10**	**10**	Pleasure doing business with you :)	**6**	**10**	**YES**	**10**
**9**	**9**	:) I dont know what to say haha but ill split it 50 50 this time for you	**18**	**10**	**YES**	**10**
**10**	**10**	Let's make some MONEY :) click in on all of them and i'll try and make it as fair as possible.	**21**	**10**	**YES**	**10**
**9**	**9**	I hope you are satisfied with the amount of money I offered you. I will offer more this time.	**19**	**10**	**YES**	**10**
**10**	**10**	I don't really have anything to say...let's split the money 10-10 again	**13**	**10**	**YES**	**10**
**10**	**10**	$10 is better than $5. Trust me, I'm a doctor haha	**11**	**10**	**YES**	**10**
**6**	**6**		**0**	**6**	**YES**	**6**
**7**	**7**	I will do exactly the same thing as I did before.	**11**	**7**	**YES**	**7**
**10**	**10**	Lets split it 11/ 9 everytime, that way we both get more money IN than OUT? sound good? I don't think you can answer me. . .	**27**	**9**	**YES**	**9**
**6**	**6**	Again I will promise $6. Please choose IN as it will maximize the profit that both of us can potentially made. I promise that I will send the full amount and if we can trust each other i will increase the amount I send in the following round. Thank you.	**50**	**6**	**YES**	**6**
**10**	**10**	Same as last time? It's only fair we earn the same amount.	**12**	**10**	**YES**	**10**
**10**	**10**	hi. i think it's best when we split it! makes it fair for everyone	**14**	**10**	**YES**	**10**
**10**	**10**	ill give u ten everytime if you choose IN then we both get ten dollars everytime we both go home with the same amount of money. again ten dollars a piece everytime go home with same amt. :)	**38**	**10**	**YES**	**10**
**10**	**10**		**0**	**10**	**YES**	**10**
**6**	**6**	want to choose in and then we take half? 10 each?	**11**	**10**	**YES**	**10**
**8**	**8**		**0**	**8**	**YES**	**8**
**10**	**10**	Let's keep going 50/50	**4**	**10**	**YES**	**10**
**7**	**7**	I promise to transfer you more money than last time.	**10**	**9**	**YES**	**9**
**9**	**9**	Hi, hope you're content with the $9	**7**	**10**	**YES**	**10**
**10**	**10**	Let's split the 20 evenly, 10-10	**6**	**10**	**YES**	**10**
**10**	**10**	Want to just split it again?	**6**	**10**	**YES**	**10**
**10**	**10**	same thing as before, we both might as well walk out with enough for gas money!	**16**	**10**	**YES**	**10**
**10**	**10**	same thing?	**2**	**10**	**YES**	**10**
**9**	**9**		**0**	**9**	**YES**	**9**

**10**	**10**	I will keep it equal like last time.	**8**	**10**	**YES**	**10**
**8**	**10**		**0**	**7**	**YES**	**9**
**9**	**9**		**0**	**9**	**YES**	**9**
**8**	**8**	Same as before Ill send you 8. We both get more $$ that way!	**14**	**8**	**YES**	**0**
**10**	**10**	Same deal.	**2**	**10**	**YES**	**10**
**10**	**10**		**0**	**10**	**YES**	**10**
**8**	**8**		**0**	**8**	**YES**	**5**
**10**	**10**	i promise to do 50/50 again	**6**	**10**	**YES**	**10**
**8**	**8**		**0**	**8**	**YES**	**8**
**8**	**8**	hey, so i just want you to know that i'll probably sent you $8 or $9! nice working with you!	**20**	**8**	**YES**	**8**
**10**	**10**	I like the way we did it last time, it works out nicely for both of us and it's fair :) ¶ Thanks for being great!	**24**	**10**	**YES**	**10**
**10**	**10**	I will be fair.	**4**	**10**	**YES**	**10**
**6**	**6**	Hi A! :)	**3**	**9**	**YES**	**6**
**8**	**8**	I'm going to do the same thing.	**7**	**8**	**YES**	**9**
**9**	**9**	I hope you're having a great day!	**7**	**8**	**YES**	**8**
**9**	**9**	Teamwork!	**1**	**10**	**YES**	**10**
**10**	**10**	I promise not to screw you out of any money and to transfer back what I say I will. If you choose in¶ we'll both benefit more! =D¶	**28**	**8**	**YES**	**8**
**10**	**10**	Don't worry, we'll evenly split the money this time, too, just like last time. I won't try to scam you or anything, because that's below me. You'll get the 10 dollars that I promise you. :)	**36**	**10**	**YES**	**10**
**10**	**10**	I'm not a risk taker and I'm not a dick. I said I'd give back ten before, and I did. We both want money. You can make $5 or $10 because I will give you ten again. yayyy money=))	**39**	**10**	**YES**	**10**
**10**	**10**	If we do this again, i'm always going to keep it equal for both of us.	**16**	**10**	**YES**	**10**
**10**	**10**	Hi, so I know it's hard to trust someone who you don't even know but I'll be I'll do my best to make things work.	**25**	**9**	**YES**	**9**
**10**	**10**	I figure we are both equally desperate for cash.	**9**	**10**	**YES**	**10**
**9**	**9**	Hi Participant A ¶ I hope you trust me due to the previous round. I will take care of you and uphold to my promises, if you take care of me. Deal? Now lets do this and make some bank! ¶ ¶ Signed, ¶ Participant B	**41**	**10**	**YES**	**10**
**8**	**8**	I'm going to offer $8 again. Hopefully you choose IN. That way we can both make a profit.	**18**	**8**	**YES**	**8**
**10**	**10**	Have you ever done this before?	**6**	**10**	**YES**	**10**
**10**	**10**		**0**	**10**	**YES**	**10**
**10**	**10**	Same thing? Seems fair? ...	**4**	**10**	**YES**	**10**
**9**	**9**	i promise i will give you what i say i will	**11**	**10**	**YES**	**10**
**10**	**10**	Thanks, glad we're both making a good amount of money! It's tough starting us off though! Wish you the best!	**20**	**10**	**YES**	**5**
**10**	**10**	Hello. Hope this doesn't sound creepy or anything. I think we should work together to get out of here with the same amount of money. I'm going to send over 10 again. :)	**33**	**10**	**YES**	**10**
**10**	**10**	I think each of us getting 10 dollars is fair. do you agree?	**13**	**10**	**YES**	**10**
**8**	**8**	same as last time :)	**5**	**8**	**YES**	**8**
**9**	**9**		**0**	**9**	**YES**	**9**
**10**	**10**	You can trust me :)	**5**	**10**	**YES**	**10**
**10**	**10**	Keep it even again	**4**	**10**	**YES**	**10**
**10**	**10**	i chose to give $10 dollars and gave you $10 in that last part. i hope we get paid	**19**	**10**	**YES**	**10**
**10**	**10**	I'm going to do the same thing as last time, 10 for you and 10 for me. We both would then walk away with 27 dollars :)	**27**	**10**	**YES**	**10**
**8**	**8**		**0**	**8**	**YES**	**8**
**9**	**9**		**0**	**9**	**YES**	**9**
**10**	**10**	Hope you like the wind....	**5**	**10**	**YES**	**10**
**8**	**8**	Were you happy with the outcome?	**6**	**9**	**YES**	**9**
**10**	**10**	Hey if you accept the $10 then we both make that everytime and thats the most mutually beneficial.	**18**	**10**	**YES**	**10**

**10**	**10**	Same thing again. We both benefit.	**6**	**10**	**YES**	**10**
**10**	**10**	hi! let's split the money 50/50 and each get 10 every time	**12**	**10**	**YES**	**10**
**7**	**7**		**0**	**7**	**YES**	**7**
**10**	**10**	Thanks for choosing IN :) hopefully if we do the same thing again we'll both make $20 each? thanks!	**19**	**10**	**YES**	**10**
**10**	**10**	Hello ¶ I wanted to make things 50/50. I don't really understand but that seemed fair to me at least	**19**	**10**	**YES**	**10**
**7**	**7**	I have no idea what to say here. This is a nice text box?	**14**	**8**	**YES**	**1**
**9**	**9**	I believe example 1 seemed the fairest for the position i was given. I did not want to be unfair however it seemed necessary to try and make a profit. I chose the smallest profit option which gave us both money in the end.	**44**	**9**	**YES**	**9**
**10**	**10**	I'll give you $10 just like before if you say "IN." ¶ It's a win-win (I get $10 instead of $0 and you get $10 instead of $5 if you were to say "OUT."	**32**	**10**	**YES**	**10**
**7**	**7**	I need a nap...	**4**	**11**	**YES**	**10**
**10**	**10**	Let's just do that same transfer again	**7**	**10**	**YES**	**10**
**8**	**8**	=]	**1**	**9**	**YES**	**9**
**10**	**10**	Hi there ¶ just trying to keep things equal and honest ¶ now let's get some solid earnings again! :)	**18**	**10**	**YES**	**10**
**8**	**8**	This is a haiku. ¶ I am glad you trusted me¶ This way we both win!	**13**	**8**	**YES**	**10**
**7**	**7**		**0**	**8**	**YES**	**8**
**9**	**9**	Please remember that if you say OUT ¶ you only get $5. I PROMISE you that I will not give you under that if you say IN ¶ I promise.	**28**	**7**	**YES**	**6**
**10**	**10**	You're in good hands. Win/win.	**5**	**10**	**YES**	**10**
**10**	**10**	I want to keep this fair and even!	**8**	**10**	**YES**	**10**
**10**	**10**		**0**	**10**	**YES**	**10**
**9**	**9**	I'm glad you trusted me and went with IN ¶ I'm gonna do the same thing again so hopefully you go with IN again :)	**24**	**9**	**YES**	**9**
**10**	**10**	hi. i liked how we did it the first time. hopefully u did too	**14**	**10**	**YES**	**0**
**10**	**10**	I will send you 10 if you select IN ¶	**10**	**10**	**YES**	**10**
**9**	**9**	$6 is the minimum offer to accept...anything higher your making more $ just off genorosity	**15**	**10**	**YES**	**6**
**10**	**10**		**0**	**10**	**YES**	**10**
**9**	**9**	Good deal! I'll up the transfer a to make it a litte more fair	**14**	**10**	**YES**	**0**
**9**	**9**	This time I'm going to promise 11 back to you ¶ and since you've seen I keep my promise ¶ when you click IN I will give you back 11 so we both walk out of here with 20. I don't break promises.	**41**	**11**	**YES**	**11**
**9**	**9**	50/50 ¶ sound good?	**3**	**10**	**YES**	**10**
**10**	**10**		**0**	**10**	**YES**	**10**
**8**	**8**	I'll give you exactly what I promise	**7**	**10**	**YES**	**10**
**6**	**7**		**0**	**10**	**YES**	**8**
**10**	**10**	just wanted to say hello :) have a nice day!	**9**	**10**	**YES**	**10**
**10**	**10**	Heyo- happy to work with you again ¶ and do the same thing.	**12**	**10**	**YES**	**10**
**10**	**10**	Hi! I'm going to split the money evenly. Have a nice day!	**12**	**10**	**YES**	**10**
**9**	**9**	Let's split it half and half ¶ ten dollars.	**8**	**10**	**YES**	**10**
**10**	**10**	Hello "A". Based on our last experiment we have established trust ¶ so thanks for making that happen! I will repeat the same steps as last time to ensure that we both get the same amount of money at our maximum level ¶ 10$ each.it makes no sense to betray each other because we just come out of this thing with less money on both parts. Lets get rich!!	**67**	**10**	**YES**	**1**
**10**	**10**	im going to offer you 10 again ¶ take it and we can profti equally	**14**	**10**	**YES**	**0**
**9**	**9**	Hey just to let you know ¶ I try my best to never lie in life and I include this experiment part of my life standard so I won't lie.	**29**	**6**	**YES**	**6**
**10**	**10**	Hey. same amount ¶ same money ¶ we both leave with 27 buck in our pocket. =]	**15**	**10**	**YES**	**10**
**10**	**10**	Teamwork + Honesty = $$$$¶	**3**	**8**	**YES**	**8**
**7**	**7**	I Hate Mondays¶ -Garfield	**4**	**17**	**YES**	**16**

**10**	**10**		**0**	**10**	**YES**	**10**
**8**	**8**	Hey! So I want to make money ¶ just as much as you do ¶ so why dont we call it even and I promise $10 ¶ you accept ¶ and we get out of here! =D Thanks	**34**	**10**	**YES**	**10**
**10**	**10**	Pay it forward. ¶ Have a great day.	**7**	**10**	**YES**	**10**
**7**	**7**		**0**	**7**	**YES**	**7**
**10**	**10**	Yay! great teamwork last time. I think we should do the same thing again this time. That way we both get the maximum amount of money. Hope that sounds good! :]	**31**	**10**	**YES**	**10**
**10**	**10**	hi hope your doing well. i plan on doing the same thing as before	**14**	**10**	**YES**	**10**
**10**	**10**	50-50 :]	**2**	**10**	**YES**	**10**
**10**	**10**	Same thing?	**2**	**10**	**YES**	**0**
**10**	**10**	I think we should do $10 each again ¶ works out best for the both of us.	**16**	**10**	**YES**	**10**
**9**	**9**	Let's do the same...It worked and we both made some money!!!!	**11**	**9**	**YES**	**9**
**10**	**10**	Hey beautiful. I hopee your having a good day. Truthfully ¶ I'll get you more money if you say IN.	**19**	**10**	**YES**	**8**
**9**	**9**	well we worked together so far- want to do it again? at least we'll both make more than $5	**19**	**7**	**YES**	**7**
**10**	**10**		**0**	**10**	**YES**	**0**
**9**	**9**	i will keep my promise!	**5**	**9**	**YES**	**9**
**10**	**10**	trust me	**2**	**20**	**YES**	**20**
**7**	**7**	I'm not quite sure what to say ¶ but hi!:)	**9**	**10**	**YES**	**0**
**9**	**9**	lets do this!	**3**	**10**	**YES**	**15**
**9**	**9**		**0**	**10**	**YES**	**10**
**9**	**9**	same deal.	**2**	**9**	**YES**	**9**
**10**	**10**		**0**	**10**	**YES**	**8**
**9**	**9**		**0**	**10**	**YES**	**10**
**9**	**9**	I'm not entirely sure what I'm supposed to say ¶ BUT point is I promise I will not jip you out of money. What I promise is what you'll get and I hope you will not jip me out of any money either :)	**43**	**9**	**YES**	**9**
**6**	**6**		**0**	**7**	**NO**	
**9**	**9**		**0**	**10**	**NO**	
**7**	**7**	I'm planning on offering the same amount so we can potentially just do the same thing as before	**18**	**7**	**NO**	
**8**	**8**	choose IN ¶ i will transfer you the promised amount of $	**11**	**9**	**NO**	
**8**	**8**		**0**	**7**	**NO**	
**10**	**10**	we need eachother to make money.	**7**	**20**	**NO**	
**8**	**8**	Hi	**1**	**7**	**NO**	
**7**	**7**		**0**	**7**	**NO**	
**9**	**9**		**0**	**8**	**NO**	
**8**	**8**	We the People of the United States of America, ¶ Inorder to form a more perfect Union, ¶ Do ordain and establish this constitution of the United States...	**24**	**8**	**NO**	
**10**	**10**		**0**	**5**	**NO**	
**6**	**6**	I'll promise to transfer whatever amount I say	**8**	**6**	**NO**	
